# Direct coupling of detergent purified human mGlu_5_ receptor to the heterotrimeric G proteins Gq and Gs

**DOI:** 10.1038/s41598-018-22729-4

**Published:** 2018-03-13

**Authors:** Chady Nasrallah, Karine Rottier, Romain Marcellin, Vincent Compan, Joan Font, Amadeu Llebaria, Jean-Philippe Pin, Jean-Louis Banères, Guillaume Lebon

**Affiliations:** 1 Institut de Génomique Fonctionnelle, Centre National de la Recherche Scientifique (CNRS), Institut National de la Santé et de la Recherche Médicale (INSERM), Université de Montpellier, F-34000 Montpellier, France; 2 MCS, Laboratory of Medicinal Chemistry, Institute for Advanced Chemistry of Catalonia (IQAC-CSIC), Barcelona, Spain; 3 Institut des Biomolécules Max Mousseron (IBMM), CNRS, Univ. Montpellier, ENSCM, Montpellier, France

## Abstract

The metabotropic glutamate (mGlu) receptors are class C G protein-coupled receptors (GPCRs) that modulate synaptic activity and plasticity throughout the mammalian brain. Signal transduction is initiated by glutamate binding to the venus flytrap domains (VFT), which initiates a conformational change that is transmitted to the conserved heptahelical domains (7TM) and results ultimately in the activation of intracellular G proteins. While both mGlu_1_ and mGlu_5_ activate Gα_q_ G-proteins, they also increase intracellular cAMP concentration through an unknown mechanism. To study directly the G protein coupling properties of the human mGlu_5_ receptor homodimer, we purified the full-length receptor, which required careful optimisation of the expression, N-glycosylation and purification. We successfully purified functional mGlu_5_ that activated the heterotrimeric G protein Gq. The high-affinity agonist-PAM VU0424465 also activated the purified receptor in the absence of an orthosteric agonist. In addition, it was found that purified mGlu_5_ was capable of activating the G protein Gs either upon stimulation with VU0424465 or glutamate, although the later induced a much weaker response. Our findings provide important mechanistic insights into mGlu_5_ G protein-dependent activity and selectivity.

## Introduction

The metabotropic glutamate (mGlu) receptors belong to class C of the large family of G protein-coupled receptors (GPCRs). mGlu receptors are localized to both synaptic and extra-synaptic sites in neurons and glia where they modulate the strength of synaptic transmission by sensing the extracellular concentration of glutamate. mGlu receptors are involved in physiological processes, such as learning and memory, and in various pathological disorders. Despite the general lack of success in recent clinical trials, mGlu receptors remain attractive drug targets for the treatment of various neuropsychiatric disorders such as anxiety and depression, neurodevelopmental disorders such as schizophrenia, and neurodegenerative disorders such as Alzheimer’s and Parkinson’s diseases^1^.

mGlu receptors are classified into 3 groups based on sequence homology and G protein signalling. Glutamate-induced stimulation of group I (mGlu_1_ and mGlu_5_) preferentially triggers Gq activation whilst group II (mGlu_2_ and mGlu_3_) and group III (mGlu_4_ and mGlu_8_) preferentially couple to Gi/o^2,3^. The molecular basis of mGlu dimer signal transduction remains poorly understood due to the lack of structures of the full-length receptor in both an active state and an inactive state. The mGlu receptors are complex allosteric machines, where glutamate activity can be potentiated by positive allosteric modulators (PAMs) or inhibited by negative allosteric modulators (NAMs)^4^. In addition, some compounds called agonist-PAMs (ago-PAMs) can bind to the allosteric binding site and display agonist activity in the absence of an orthosteric agonist such as glutamate.

From a molecular perspective, mGlu and other class C receptors are unusual compared to the majority of GPCRs. They are obligatory dimers, with dimerization being essential for their function^3,5^. Each mGlu monomer contains a large extracellular domain (ECD) composed of the venus flytrap domain (VFT) and a cysteine-rich domain (CRD). The VFT is a bi-lobed structure, with a cleft between the lobes that forms the orthosteric-binding site. The CRD links the VFT to the heptahelical transmembrane domain (7TM), which then terminates in the C-terminal domain. Some allosteric modulators are known to bind to the 7TM domain. It is well established that the C-terminal region of mGlu receptors is unstructured, although it is important for signal modulation^6^.

An additional level of complexity is added to mGlu receptor function when functional selectivity is considered. Recent studies have reported biased allosteric modulation of mGlu receptors, in particular for mGlu_5_^7^, although these studies were complicated by the fact that only changes in downstream effectors were analysed. Signalling of the mGlu_5_ receptor in HEK cells or in neurons induced by the agonist 3,4-dihydroxyphenylglycol (DHPG), in the presence or absence of a variety of PAMs, or by using an ago-PAM alone^8^, induced the activation of different signalling pathways^7^, but all through Gq. Additionally, agonist-induced mGlu_5_ increased cAMP levels in transfected cells^9^, although it was unclear in this study whether this was by direct activation of Gs by mGlu_5_. The ligand-dependent functional selectivity highlights the complexity and diversity of mGlu_5_ signal transduction. It also emphasizes the importance of understanding the molecular basis for ligand-receptor interactions and how these drive conformational changes that lead to distinct signalling outputs. Developing drugs targeting the allosteric binding site in order to achieve functional selectivity may be highly beneficial in reducing side effects. However, this remains a challenging task due mainly to the lack of understanding of the structural basis of mGlu receptor signal transduction.

Structures have been determined of isolated domains of class C GPCRs, which includes VFTs and ECDs of several mGlu receptor subtypes, the calcium receptor, the GABA_B_ receptor and the taste receptor^10–14^. In addition, the structures of the 7TM domains of mGlu_1_ and mGlu_5_ have been determined by X-ray crystallography^15,16^, although these are both in an inactive state. The structure of a full-length mGlu receptor has yet to be determined. Thus it remains unclear how conformational changes are propagated from the VFT to the 7TM^17,18^ and how functional selectivity is attained. Functional characterization of the detergent purified full-length mGlu receptor dimer will be essential to understand these phenomena.

Here we have investigated the expression, stability and purification of the human mGlu_5_. The wild-type human mGlu_5_ receptor was expressed in insect cells and purified to homogeneity, providing sufficient monodisperse receptor to initiate functional studies. The purified receptor was functional and activated the G protein Gq upon binding of either the endogenous orthosteric agonist glutamate or the synthetic high affinity ago-PAM, VU0424465. Finally, we demonstrated that VU0424465 induced mGlu_5_-dependent Gs activation in HEK293 cells and also the purified receptor in detergent bound Gs. Thus the purified functional full-length mGlu_5_ receptor produced within this study represents the first step towards its structure determination.

## Results

### Defining a minimum human mGlu_5_ construct

**Figure 1:**
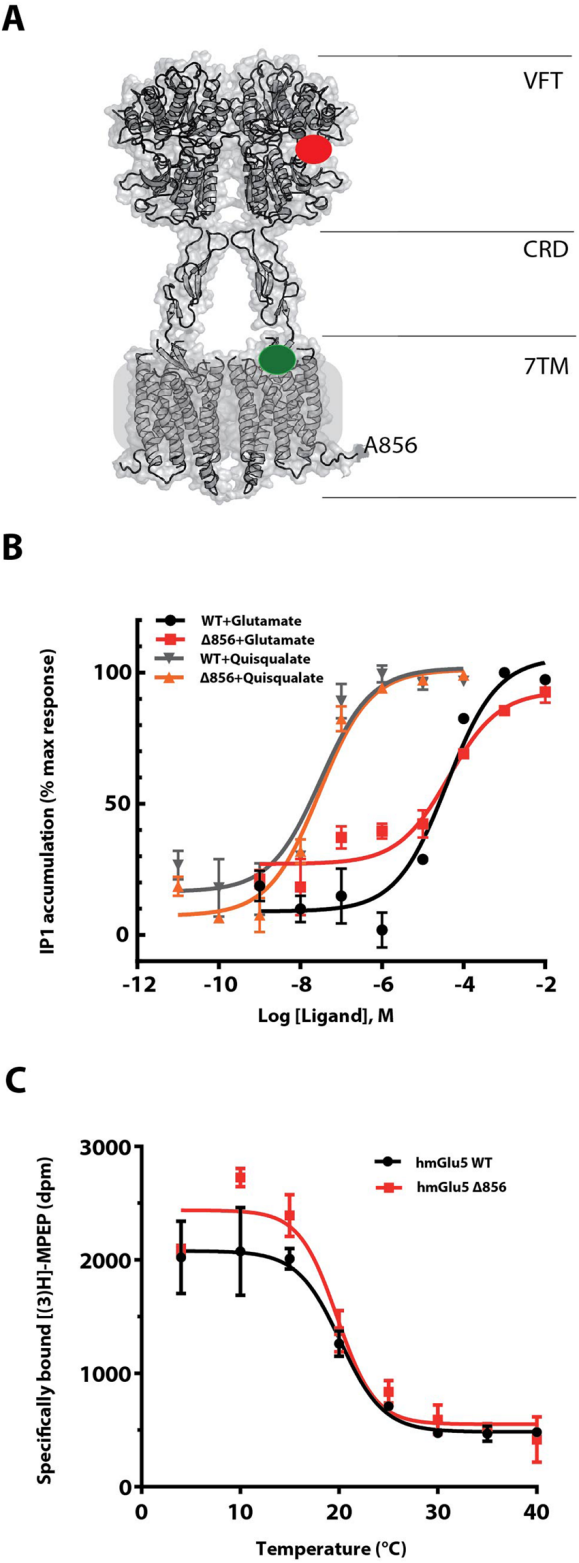
Functional C-terminally truncated SNAP-mGlu_5_ receptor dimer. (**A**) 3-Dimensional model of the human mGlu_5_ receptor truncated after residue Ala856. The truncated receptor is composed of all segments required for effective G protein signaling, including the Venus Flytrap domain (VFT), Cysteine-Rich Domain (CRD) and the heptahelical transmembrane domain (7TM). The red and green spheres represent the orthosteric and allosteric binding sites, respectively. (**B**) Glutamate- and quisqualate-induced IP1 production in HEK293 cells transiently transfected with SNAP-Tag full-length human mGlu_5_ receptor (black and grey curves) or SNAP-Tag C-terminal mGlu_5_-Δ856 receptor (red and orange curves), respectively. (**C**) Thermal stability of the full-length wild-type mGlu_5_ and mGlu_5_-Δ856 [^3^H]-MPEP-bound receptors. Data points for both assays represent the mean ± SEM of three independent experiments.

The C-terminus of class C GPCRs is considered to be unstructured^19^ so in order to minimize receptor flexibility, we investigated the minimum length of the C-terminal tail required for preserving Gq protein activation. A series of C-terminal truncations were designed by introducing stop codons that shortened the C-terminus from residue L865 up to residue N832, which corresponds to the intracellular end of transmembrane helix 7 (TM7). Gq activation was then examined for each of the mutants by measuring phosphoinositide (PI) breakdown using the Cisbio IP-One® assay (Supplementary Figs 1 and 2). The data show that the C terminal tail of mGlu_5_ can be truncated up to residue K851 without significant reduction of glutamate potency for Gq activation. Deleting further C-terminal amino acid residues impaired Gq activation. Although mGlu_5_-Δ851 had the minimum length of C terminus that allowed signalling, we selected mGlu_5_-Δ856 for future work to ensure a sufficient length to allow for the formation of helix 8 as observed in class A GPCRs^20^. The mutant hmGlu_5_-ΔCter-A856 had a pEC_50_ of 4.35 ± 0.06 (n = 5) for glutamate and 7.34 ± 0.18 (n = 5) for quisqualate (Fig. 1A and Supplementary 2). These values were similar to those obtained for the full-length wild-type (WT) mGlu_5_ receptor with pEC_50_s of 4.48 ± 0.05 (n = 5) for glutamate and 7.51 ± 0.15 (n = 5) for quisqualate (Fig. 1B). The thermal stabilities of the truncated construct (SNAP-mGlu_5_-Δ856) and the full-length construct (SNAP-mGlu_5_-WT) were also determined. Both constructs display an similar apparent T_m_ value in agreement with previously published work (WT, 19.95 ± 0.73 °C (n = 6); mGlu_5_-Δ856 19.87 ± 0.67 °C (n = 6); Fig. 1C)^16^. Therefore, construct mGlu_5_-Δ856 is a fully functional GPCR with all structural features required for dimerization and activation of the human mGlu_5_ receptor, and it was used for all further biochemical characterization and purification.

### Characterization of N-linked glycosylation

**Figure 2:**
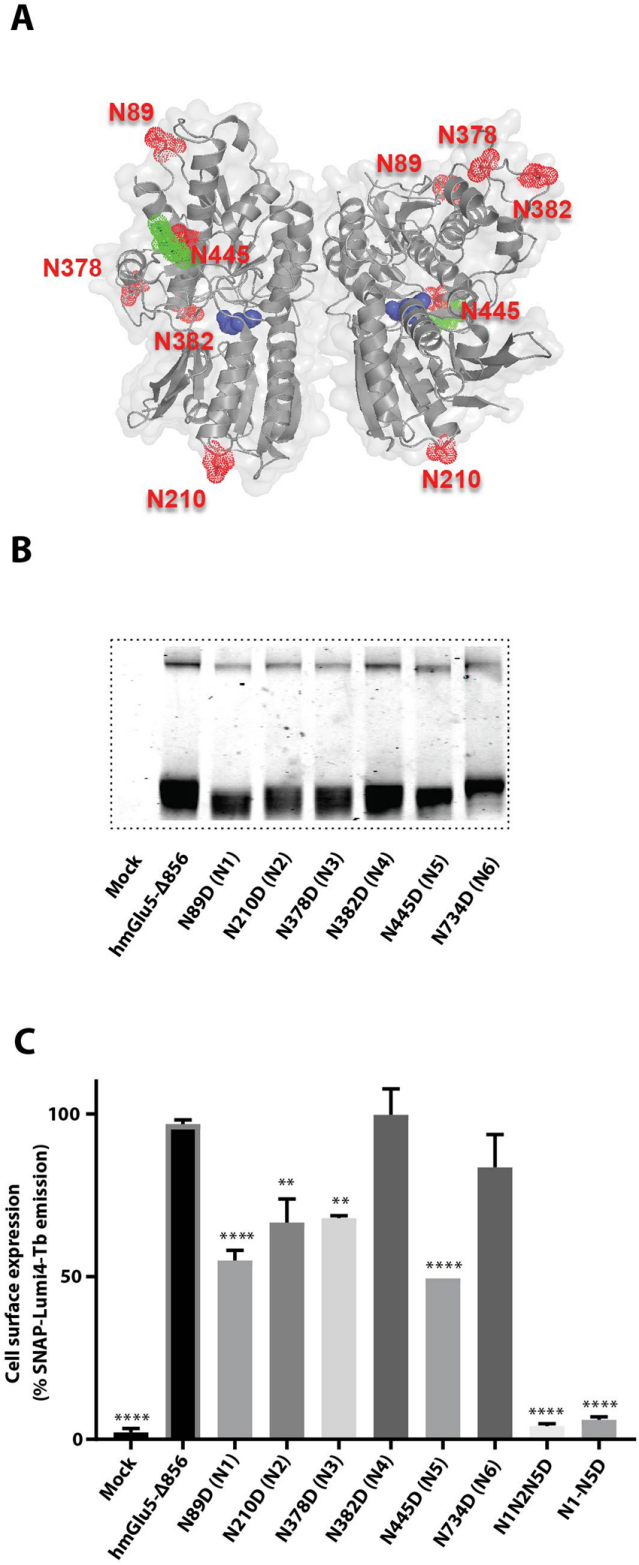
Characterization of the SNAP-mGlu_5_-∆856 N-glycosylation profile in HEK293 cells. (**A**) The five mGlu_5_ N-linked glycosylation sites are highlighted as surface spheres (red) and the sugar moieties in green on the cartoon structure (grey) of the VFT (code pdb 3LMK). Atoms in the glutamate molecules bound to the VFT are represented as blue spheres. (**B**) SDS-PAGE gel migration of monomeric SNAP-mGlu_5_-Δ856 and different N-glycosylation mutants. Samples were treated with reducing agent (DTT). (**C**) Cell surface expression of the fluorescently labeled SNAP-mGlu_5_-Δ856 and different N-glycosylation mutants using SNAP-Lumi4Tb. Gel is representative of one experiment repeated at least two time. Expression profiles are representative of at least three separate experiments. Dunnett’s test as part of one*-*way ANOVA was used for comparison with hmGlu_5_-Δ856 set as reference level.

The human mGlu_5_ amino acid sequence contains 6 potential N-glycosylation NX(S/T) consensus sites in regions of the receptor predicted to be on the extracellular surface. Five of these sites are in the large VFT domain (Fig. 2A) and one additional site is in the second extracellular loop (EL) of the 7TM. The heterogeneity of N-glycosylated receptors can hinder their crystallization, but N-glycosylation may also be important for expression of functional receptor at the cell surface. We explored the contribution of these sites to cell surface expression of well-folded receptor.

Six mutants of SNAP-mGlu_5_-Δ856 were generated in which each of the native N-glycosylation sites was mutated from NxS/T to DxS/T. These constructs were then expressed transiently in HEK293 cells and the extracellular SNAP tag labelled with a cell-impermeant red fluorophore. The SNAP-tagged receptor was then extracted from the cell using suitable detergents and the non-purified material was analysed by SDS-PAGE. The SNAP-mGlu_5_-Δ856 receptor migrated predominantly as dimers (apparent molecular weight 180–200 kDa) under non-reducing conditions and as a monomer (apparent molecular weight 90–100 kDa) under reducing conditions (Supplementary Fig. 3). Although both monomeric and dimeric receptor could be visualized on an SDS-PAGE gel, we based our analysis on the gel mobility shifts of the monomeric species formed under reducing conditions (plus DTT) since small changes in molecular weight were more obvious. The data show a small decrease in molecular weight of the monomeric receptor when each of the N-glycosylation sites N1-N5 were mutated (Fig. 2B), suggesting they are involved in glycosylation of the WT receptor. However, mutation of the N6 site localized in the 7TM domain did not appear to be N-glycosylated since the apparent molecular weight of the mutant was similar to the wild type receptor.

The effect of these mutations on the cell surface expression of SNAP-mGlu_5_-Δ856 receptor was determined for the five N-glycosylation site mutants N1-N5. Mutating N1, N2, N3 and N5 significantly impairs cell surface expression of the receptor (Fig. 2C). Only the mutation N382D (N4) did not show any reduction in expression level at the cell surface, although the decrease in apparent molecular weight suggested that this site is N-glycosylated. In efforts to decrease further the amount of N-glycan on the receptor, one mutant was constructed where three glycosylation sites (N1N2N5) were mutated and another mutant combined all 5 N-glycosylation sites mutated (N1-N5). None of the triple (N1N2N5) or quintuple mutants (N1-N5) were detected at the cell surface after fluorescent labelling of the SNAP-tag (Fig. 2C). Taken together, these results suggest that all 5 N-glycosylation sites localized in the VFT are N-glycosylated in HEK293 and Sf9 cells and that N-glycosylation of at least four sites plays a critical role in ensuring cell surface expression of the receptor.

### Detergent solubilisation and stability of mGlu_5_-Δ856

Structural characterization of GPCRs is complicated by their lack of stability in detergent and therefore they are difficult to purify in a functional state^21^. Therefore, it was important to identify a suitable detergent that maintained mGlu_5_ in a functional and stable state upon solubilisation. One way to measure the relative quantity of detergent-solubilized receptor as well as its thermal stability is to use a radioligand-binding assay^22^. For this purpose, we used the ‘super plus’ format radioligand-binding assay that consists of equilibrating the receptor with radiolabeled ligand before performing detergent solubilisation (see material methods section)^22,23^. This allows the determination of an apparent melting temperature (apparent T_m_) defined as the temperature at which 50% of the solubilized receptor can still bind the radioligand. The apparent T_m_ is a reliable indicator of the stability of the unpurified receptor in detergent solution.

**Figure 3:**
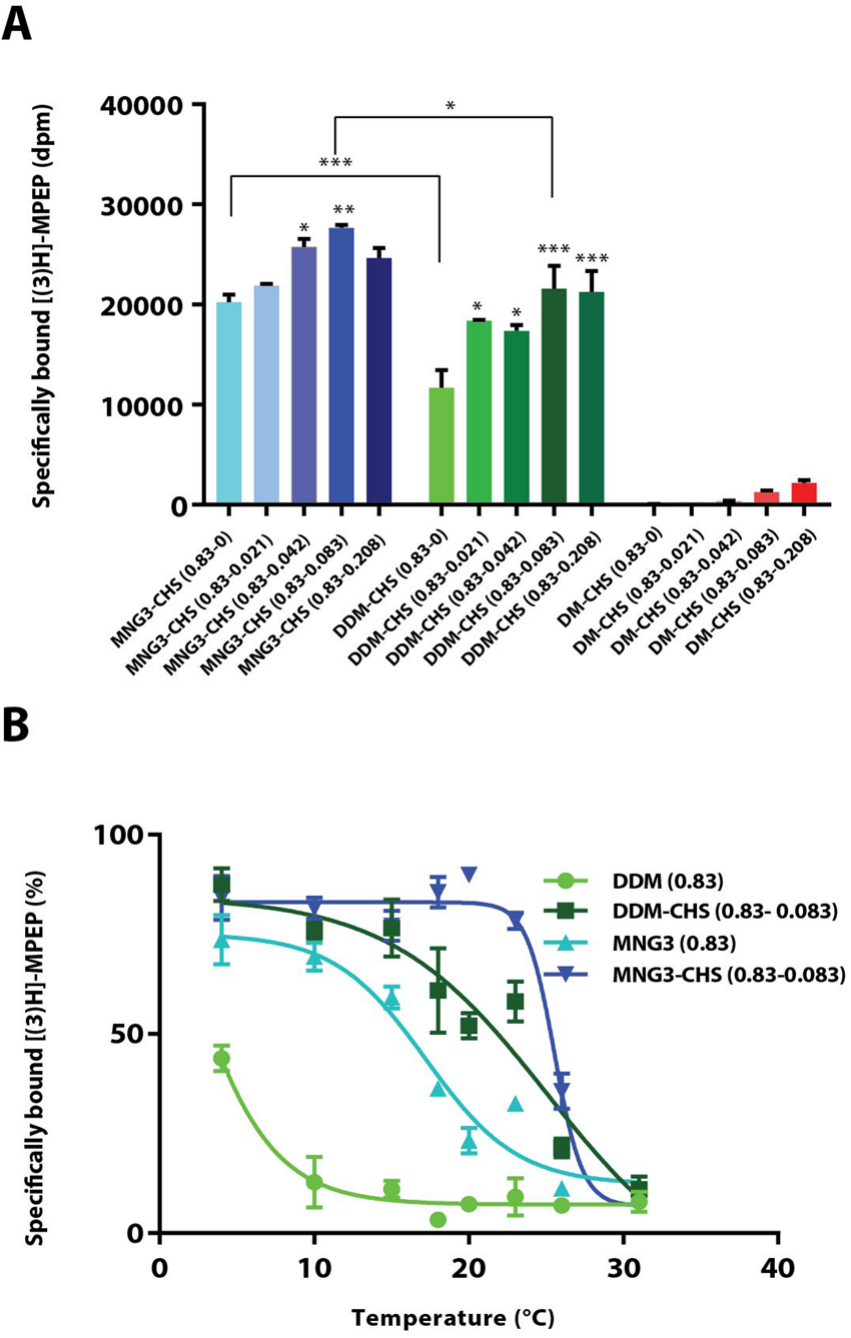
Detergent solubilisation and thermal stability of mGlu_5_-Δ856 [^3^H]-MPEP-bound receptor in insect cells. (**A**) Quantification of unpurified mGlu_5_-Δ856 receptor solubilized at 4 °C using [^3^H]-MPEP binding assay measured in a series of maltoside-based detergents including lauryl maltose neopentyl glycol-3 (MNG3), n-Dodecyl-β-D-Maltoside (DDM) and n-Decyl-β-D-Maltoside (DM) supplemented with different ratio of cholesteryl hemisuccinate (CHS). (**B**) Thermal stability of the mGlu_5_-Δ856 [^3^H]-MPEP-bound receptor solubilized in four different conditions; DDM (0.83%), DDM-CHS (0.83–0.083%), MNG3 (0.83%) and MNG3-CHS (0.83–0.083%). Data points for both assays (**A,B**) represent the mean ± SEM of three independent experiments. Dunnett’s test as part of one*-*way ANOVA was used for comparison with MNG3-CHS (0.83–0) and DDM-CHS (0.83–0) set as reference level for MNG3 and DDM supplemented with CHS, respectively.

mGlu_5_-Δ856 was expressed in insect cells and the high-affinity radiolabeled NAM [^3^H]-MPEP that binds to the 7TM domain was used to monitor the percentage of functional receptor after detergent solubilisation. We first tested two mild non-ionic detergents that have been used successfully for the isolation and purification of many GPCRs, namely *n*-dodecyl-β-D-maltoside (DDM) and maltose-neopentyl glycol-3^24^ (MNG3). In addition, we also tested the shorter alkyl chain homologue decyl-β-D-maltoside (DM). Detergent solubilisation conditions were optimised further by complementing the selected detergents with cholesteryl hemisuccinate (CHS), a cholesterol derivative used to further stabilise GPCRs. Detergents were first evaluated for their ability to solubilise mGlu_5_ in a functional form that could still bind [^3^H]-MPEP at 4 °C (Fig. 3A). Our results showed clearly that the highest amount of solubilized functional mGlu_5_-Δ856 receptor was with MNG3, whereas DDM was two-fold less efficient. The detergents were then supplemented with CHS to give a final concentration ranging from 0.04% to 0.08%. The quantity of solubilised receptor increased slightly when MNG3 was supplemented with CHS and attained a maximum yield at 0.08% CHS. Adding CHS also significantly improved the amount of solubilised receptor in DDM. We found that a concentration of CHS of 0.08% was optimal for solubilising the mGlu_5_ receptor dimer. mGlu_5_ receptor was not stable after solubilisation in DM even in the presence of CHS.

To define the best detergents for solubilisation and purification, the thermostability of mGlu_5_ bound to [^3^H]-MPEP was determined in 0.8% MNG3 and 0.8% DDM, in the presence or absence of 0.08% CHS (Fig. 3B). The apparent T_m_ of mGlu_5_ receptor solubilized in MNG3 was 18 ± 0.3 °C (n = 3), but the receptor was too unstable in DDM for an apparent T_m_ to be measured. Adding CHS significantly increased the stability of mGlu_5_ in both detergents, where it had an apparent T_m_ of 21 ± 1.5 °C (n = 3) in DDM/CHS and an apparent T_m_ of 25 ± 0.1 °C (n = 3) in MNG3/CHS. As MNG3/CHS gave the best stability for [^3^H]-MPEP-bound mGlu_5_-Δ856, we used these conditions for solubilisation and purification.

### Expression and purification of mGlu_5_-Δ856 from Sf9 cells

**Figure 4:**
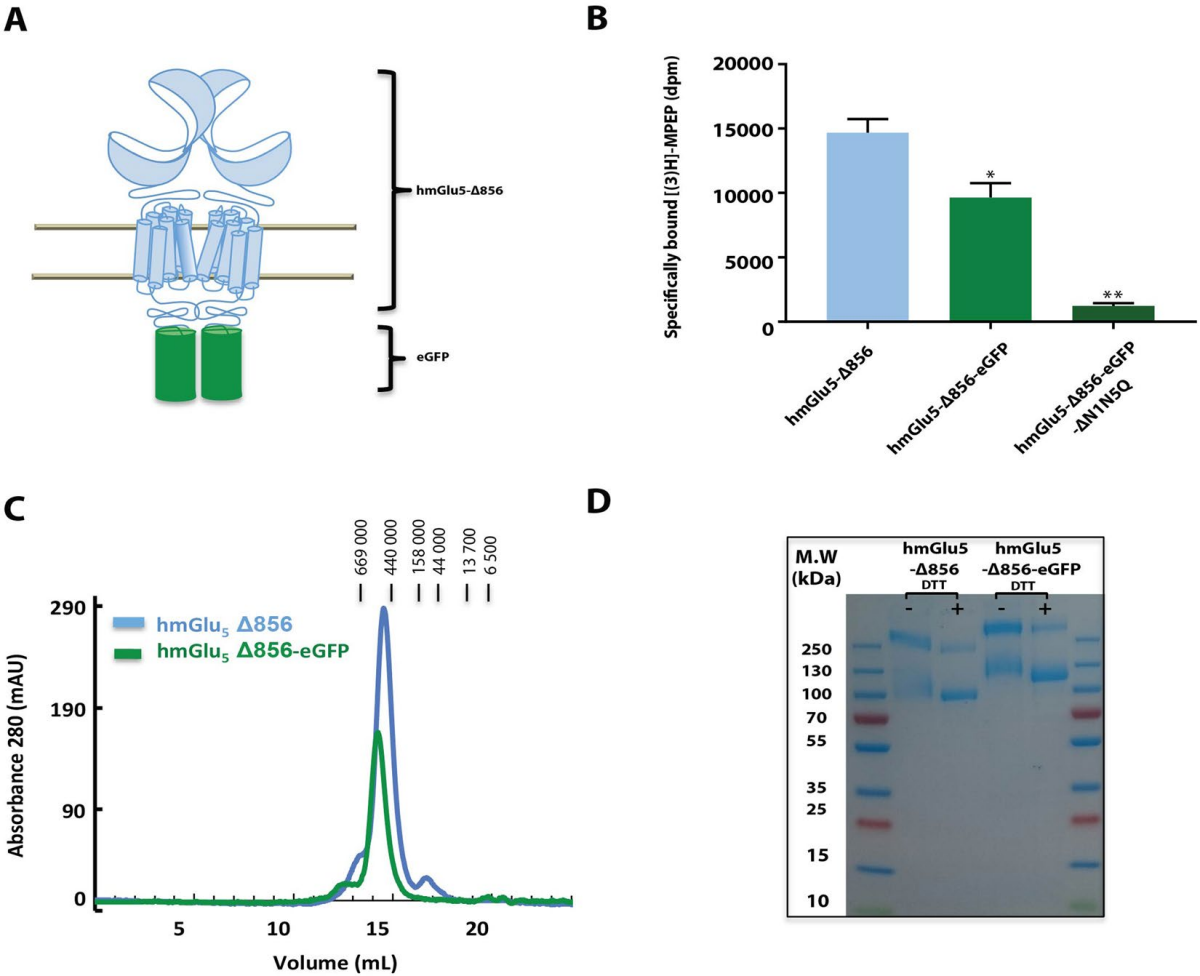
Purification of the dimer mGlu_5_ -∆856 produced in SF9 cells. (**A**) Cartoon representation of the dimer human mGlu_5_ receptor (light blue) with the fusion protein eGFP (green) replacing the c-terminal domain at position 856. (**B**) Quantification of unpurified mGlu_5_ receptor using [^3^H]-MPEP binding assay, measured for mGlu_5_-Δ856, mGlu_5_-Δ856-eGFP and mGlu_5_-Δ856-eGFP (N1-N5). Dunnett’s test as part of one*-*way ANOVA was used for comparison with mGlu_5_-Δ856 set as reference level. (**C**) Size exclusion chromatography profile for both constructs, mGlu_5_-Δ856 (Ve = 14.5 mL) and mGlu_5_-Δ856-eGFP (Ve = 14.2 mL) using superpose 6 increase. (**D**) Coomassie-blue stained SDS-PAGE gel of mGlu_5_-Δ856 and mGlu_5_-Δ856-eGFP. The gel shown is representative of three independent experiments.

Large-scale production of mGlu_5_-Δ856 was performed in Sf9 cells using the baculovirus expression system. This system was used to express most of the crystallized GPCRs to date, because it often gives good yields and it can perform post-translational modifications such as N-glycosylation^25^. We first optimized the purification of mGlu_5_-Δ856 fused at the C-terminus to eGFP (Fig. 4A). mGlu_5_-Δ856-eGFP showed similar pharmacology to the wild-type receptor as assessed using the [^3^H]-MPEP binding assays (Fig. 4B). As for HEK293 cells, glycosylation are important for receptor expression. We have tested expression of mGlu_5_ ΔN1-N5 mutants in Sf9 insect cells. Mutating all glycosylation sites abolishes receptor expression (Fig. 4B). Purification of mGlu_5_-Δ856-eGFP was achieved on a Ni^2+^-affinity column followed by size exclusion chromatography (SEC) in MNG3/CHS (Fig. 4C). The gel filtration profile (Superose 6 increase, GE Healthcare) of the purified receptor was symmetrical, which suggested that the purified receptor was monodisperse. Non-reducing SDS-PAGE identified a major band with an apparent molecular weight (MW) of ~250 kDa and a minor band of 125 kDa, which corresponds well to the theoretical MW of the mGlu_5_-eGFP dimer (249,568 Da) and monomer (124,784 Da) (Fig. 4D). The identity of the protein bands on SDS-PAGE was confirmed by mass spectrometry (Supplementary Fig. 4). Both bands yielded fifteen peptide segments that matched the sequence of mGlu_5_-eGFP with a sequence coverage of 60%. Although the SEC profile did not show the presence of any monomer, a minor band corresponding to the monomer was observed by SDS-PAGE, possibly due to dissociation caused by SDS. Treatment of the purified sample with reducing reagents to disrupt the dimer increased the intensity of the monomeric species migrating at 125 kDa compared to the non-reduced sample (Fig. 4D).

We initially mutated only one glycosylation site (N445) to alanine in this construct as sugar moiety was obvious in the available PDB structure of the mGlu_5_ VFT (3LMK) (Fig. 2A). Indeed, mutating multiple glycosylation sites was not possible due to severe decrease in expression level as we demonstrate above (Fig. 2C). Treatment of the purified receptor with peptide-*N*-glucosidase F (PNGase F) overnight followed by SEC resulted in the purification of the deglycosylated receptor (Supplementary Fig. 4C). A final yield of 0.05 mg of purified mGlu_5_-eGFP protein per liter of Sf9 insect cell culture was obtained. We also purified another construct, mGlu_5_-Δ856, devoid of eGFP at the C-terminus (Supplementary Fig. 1C) using the same protocol except that an additional purification step was included (affinity chromatography using an anti-FLAG tag antibody resin). We finally obtained 0.04 mg of highly purified mGlu_5_-Δ856 per liter of Sf9 (Fig. 4), which was furthermore characterized *in vitro* using a G protein activation assay.

### Functional characterization of purified mGlu_5_ receptor

**Figure 5:**
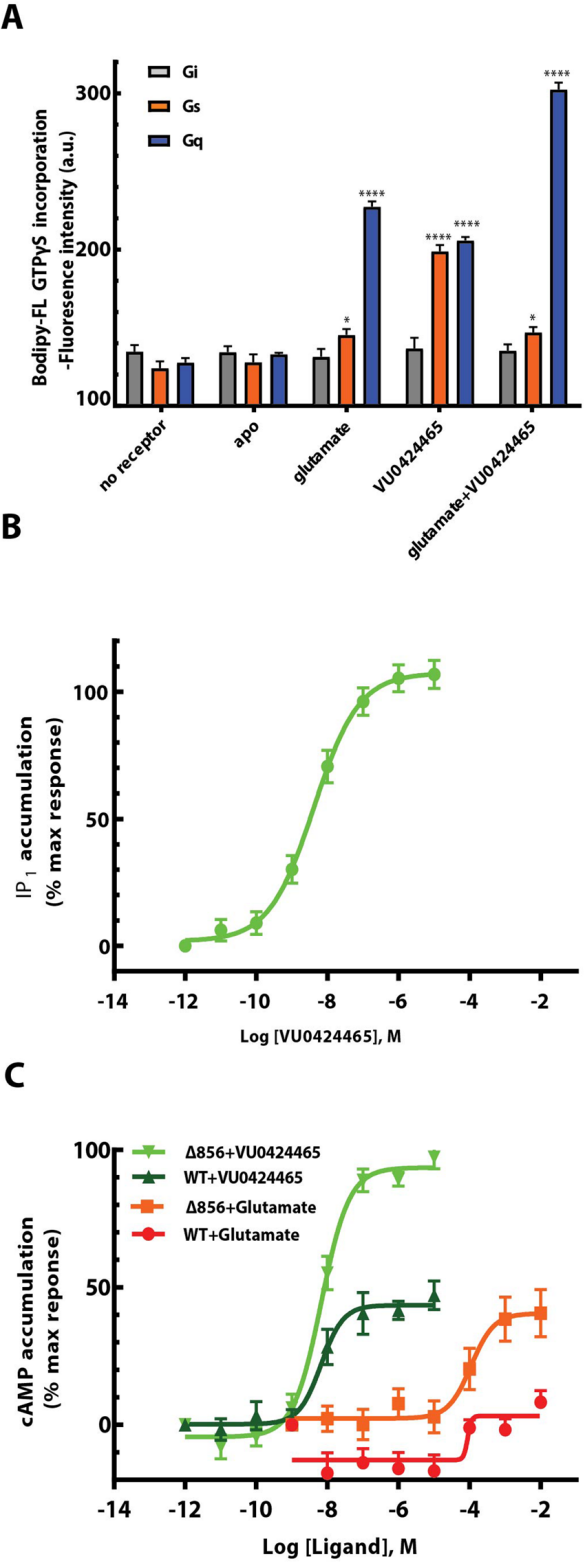
Glutamate and VU0424465 trigger mGlu_5_-dependent Gs and Gq activation. (**A**) GTPγS fluorescence binding experiments were carried out within detergent purified receptor induced by ligand activation. The fluorescence baseline is estimated based on the receptor alone without any ligands. Data points represent average of three independent measurements with SEM. Dunnett’s test as part of one*-*way ANOVA was used for comparison with the apo condition set as reference level. (**B**) mGlu_5_-dependent Gq activation was measured using the IP one cell-based assay kit (Cisbio) in HEK293 cells and in the presence of VU0424465 with a pEC_50_ of 8.53 ± 0.13 (n = 4). (**C**) mGlu_5_-dependent Gs activation was measured using the cAMP cell-based assay kit (Cisbio) in HEK293 cells and in the presence of glutamate and VU0424465, independently. The dose response curves show a positive response for VU0424465 at the truncated mGlu_5_-Δ856 (light green, pEC_50_ of 8.17 ± 0.11; n = 4) and WT human mGlu_5_ (Dark green, pEC_50_ of 7.80 ± 0.50; n = 3) curve. A weaker activation is observed for glutamate at the truncated mGlu_5_-Δ856 (orange, pEC_50_ of 3.86 ± 0.20; n = 3) and WT human mGlu_5_ (red, pEC_50_ of 3.36 ± 0.72; n = 3) curve. Data points represent the average of triplicate measurements from at least three independent experiments with SEM.

Purified mGlu_5_-Δ856 was tested for its capacity to activate the purified heterotrimeric G protein Gq using the fluorescent Bodipy-FL GTPγS analog^26^. The native agonist glutamate induced recruitment and activation of Gα_q_β_1_γ_2_ as demonstrated by the increase in Bodipy-FL GTPγS fluorescence compared to the control conditions (Fig. 5A). The ago-PAM VU0424465^27^ produced a strong response similar to glutamate and displays a pEC_50_ of 8.53 ± 0.13 (n = 4) in HEK293 cells (Fig. 5B). The purification protocol developed here excluded the presence of glutamate, so that these data confirmed the capacity of VU0424465 to directly activate the receptor. Finally, the combination of glutamate and VU0424465 increased the maximum response by 30%, supporting the allosteric role of VU0424465.

We also investigated the G protein selectivity of purified mGlu_5_-Δ856 by testing its ability to activate purified Gs and Gi. We could not measure any Gi activation upon glutamate or VU0424464 stimulation of mGlu_5_-Δ856 (Fig. 5A). In contrast, strong activation of Gs was observed, with a fluorescence signal intensity similar to that observed for the activation of Gq by VU0424464. We confirmed this finding by demonstrating a strong cAMP dose response induced by VU0424465 as measured in HEK293 cell line expressing mGlu_5_-Δ856 (Fig. 5C). VU0424465 displayed a pEC_50_ of 8.17 ± 0.11 (n = 4) for the Gs signalling pathway similar to Gq activation (pEC_50_ of 8.53 ± 0.13 (n = 4)) (Fig. 5B). Glutamate stimulation of purified mGlu_5_-Δ856 induced only partial activation of Gs G protein (Fig. 5A). Accordingly a weak but significant cAMP production was detected upon mGlu_5_ receptor stimulation at high concentration with a pEC_50_ of 3.86 ± 0.20 (n = 3) (Fig. 5C). We have also tested WT human mGlu_5_ receptor that includes the complete c-terminus for its capacity to activate Gs G protein (Fig. 5C). For both ligands, VU0424465 and Glutamate, cAMP production is significantly impaired compared to mGlu_5_-Δ856. To our surprise, the combination of glutamate and VU0424465 bound to detergent-purified mGlu_5_-Δ856 activated Gq, but not Gs. When mGlu_5_-Δ856 was reconstituted into a membrane like environment such as bicelles, glutamate and VU0424465 bound to mGlu_5_-Δ856 resulted in the activation of Gs (Supplementary Fig. 5). In addition, Gq activation was also significantly increased for receptor reconstituted in bicelles. Thus it is likely that lipids play a key role in mGlu_5_ functionality and stability.

## Discussion

Here we describe the biochemical characterization and protein engineering leading to the purification of an intact and functional mGlu_5_ receptor dimer. Purified mGlu_5_-Δ856 contains all the domains required for its function as verified by a G protein activation assay and we demonstrate for the first time direct coupling of human mGlu_5_ to the heterotrimeric G protein Gs. The purified receptor comprised the ECD and the 7TM domain, while the C-terminal tail was shortened to reduce unstructured regions^19^. Removal of 324 amino acids (S857-L1180) from the intracellular C-terminal domain did not impair Gq protein signalling or receptor stability (Fig. 1). Indeed the selected construct remained fully functional and the apparent T_m_ of mGlu_5_-Δ856 of 20 °C was identical to the WT mGlu_5_, which implies that the C-terminal domain does not participate in maintaining the overall fold of the receptor.

When considering the homogeneity of the purified receptor, an obvious parameter that needs to be taken into account is *N-*glycosylation. Like many other eukaryotic membrane proteins, mGlu_5_-Δ856 requires post-translational *N-*glycosylation for trafficking of the functional receptor to the cell surface, precluding the mutation of all the glycosylation sites. Removal of individual N-glycosylation sites significantly impaired cell surface expression, with the exception of the mutants for N382 in the VFT and N734 in ECL2 (Fig. 2C). A fully unglycosylated mGlu_5_ receptor mutant could not reach the cell surface when expressed either in HEK293 or SF9 cells (Figs 2C and 4B). This finding highlights the importance of preserving N-glycosylation for expression of mGlu_5_-Δ856 in the plasma membrane. N-Glycosylation is present on other class C GPCRs and its role is variable depending on the receptor. For example, N-glycosylation is essential for cell surface expression of the GABA_B_ receptor^28^, whereas N-glycosylation of the human mGlu_1_ receptor is not required for either receptor expression or function^29^.

The stability of mGlu_5_-Δ856 in detergent was further investigated. Several detergents were tested for their ability to maintain mGlu_5_-Δ856 in a functional state, including MNG3^24^ and DDM, both containing C12 poly-unsaturated hydrophobic aliphatic chains, and DM a C10 molecule known to be less stabilizing for GPCRs. MNG3 stands out as the optimal detergent to extract receptor from the plasma membrane, because it imparted on the receptor a higher stability when compared to DDM or DM. MNG3 was first demonstrated to provide stability in the absence of CHS. DDM alone was not sufficient to maintain the fold of the receptor in solution and must be complemented with CHS for functional solubilisation of human mGlu_5_ receptor (Fig. 3). Further complementing MNG3 detergents with CHS increased the apparent T_m_ by 7 °C at 10:1 MNG3:CHS ratio (Fig. 3B). Not surprisingly, the smaller detergent DM (C10) failed to maintain mGlu_5_ in a functional state. These data show that mGlu_5_-Δ856 is poorly stable in detergent.

The mGlu_5_-Δ856-eGFP fusion was used to facilitate setting up the purification protocol, in a manner similar to other membrane proteins^30^, and successfully led to pure mGlu_5_ receptor dimer (Fig. 4). SDS-PAGE under non-reducing conditions revealed an intense band that migrated at the expected molecular weight for an mGlu_5_-Δ856-eGFP dimer. Disulphide bonds between conserved cysteine residues in lobe I of each VFT protomer are essential for dimerization^31^ and there was a clear shift to the monomeric state when mGlu_5_-Δ856 was treated with a reducing reagent. In addition, the observed SEC profile is monodisperse and of a relative molecular weight consistent with a dimer (Fig. 4C).

Finally, we investigated the functionality of purified mGlu_5_-Δ856 through its ability to activate purified G proteins. Activation of the heterotrimeric G protein Gq upon glutamate or VU0424465 stimulation showed that the purified receptor was fully functional and was in a native conformation (Fig. 5). The combination of both ligands further increased the activation of Gq, which suggests that VU0424465 further stabilizes the active conformation induced upon glutamate binding and highlights the allosteric properties of the purified mGlu_5_ dimer. Surprisingly, this study also demonstrated the capacity of mGlu_5_ to directly activate Gs upon binding of VU0424465 to the 7TM domain^27^. It was previously reported that Group I mGlu receptors couple to Gs in cell-based assay^9^. However, there was no clear evidence demonstrating the direct coupling of Gs to mGlu_5_.

Here, we demonstrate the direct coupling of detergent purified mGlu_5_ receptor to Gs that is further confirmed in HEK293 cell expressing mGlu_5_-Δ856 receptor (Fig. 5C). In addition, the length of the receptor C terminus directly impact Gs coupling for both ligand, glutamate and VU0424465. However mGlu_5_-Gs coupling appears to be much less efficient compared to Gq coupling (Fig. 5). Purified mGlu_5_ receptor dimer in the presence of both glutamate and VU0424465 efficiently activated only Gq and not Gs. This suggests that they are different active conformations of mGlu_5_ and this supports in part the G protein selectivity of the receptor^32^. Clearly, the conserved recognition sequences for Gs coupling will differ to those for Gq and will affect the strength of G protein coupling^33^. However, it is difficult to speculate on mGlu_5_ residues that directly interact with Gs or Gq in the absence of structural data. The observed VU0424465-induced stimulation of Gs might be explained by the high-affinity and strong capacity of the molecule to stabilize the active conformation of the 7TM independently of a selective quaternary conformation of the receptor dimer, a fact compatible with the weak Gs activation observed for glutamate. It is tempting to hypothesize that, in the absence of glutamate, the 7TM unit of the mGlu_5_ receptor dimer may function like a class A GPCR^34^. In theory, it is possible that the active conformation of the 7TM bound to the high affinity PAM VU0424465 may be different from the active conformation induced upon co-binding of VU0424465 and glutamate. Thus the molecular determinants of G protein selectivity requires a more in-depth characterization, taking into account the contribution of the receptor dimer in sampling specific conformations of the individual 7TM domains.

In conclusion, the purified mGlu_5_ receptor represents an excellent tool for investigating the molecular basis of class C GPCR signal transduction. We demonstrate that mGlu_5_ receptor is able to directly activate Gs, which should be considered as an alternative-signalling pathway when developing new drugs targeting mGlu_5_. Indeed the very potent and efficacious action of VU0424465 highlights the need for a systematic characterization of the action of allosteric modulators, not only on the main signalling cascade of the receptor, but also on other cellular pathways. These alternative pathways may either increase the beneficial effect of a drug or, in contrast, lead to deleterious side effects. Further characterization of the selectivity of mGlu_5_ G protein signalling is required, not only in heterologous expression system but also in neurons. The findings reported here might have implications for the future development of drugs targeting mGlu_5_ receptor. For example, VU0424465 has been demonstrated to display some side effects such as inducing seizures in small animals^27^. These undesired effects have been associated with its self-agonist activity and could be related to biased signalling properties of the compounds^35^. A high-resolution structure of the intact receptor is required to fully understand the structural features of the mGlu_5_ receptor dimer, its mode of activation and to decipher its G protein coupling selectivity.

## Methods

### Materials

Tritiated 2-methyl-6-(phenylethynyl)pyridine hydrochloride [^3^H] MPEP was purchased from the American Radiolabeled Chemicals incorporation (ARC), glutamate from Sigma Aldrich, quisqualate and MPEP from Tocris. Sf9 cells adapted in SF-900 II SFM and EX-CELL-420 medium were ordered from Sigma Aldrich, lipofectamine 2000 and DMEM medium from Life Technologies. Detergents were ordered from Anatrace. SNAP-Lumi4-Tb and SNAP Red were obtained from Cisbio Bioassays. Bodipy-FL GTPγS was purchased from ThermoFisher. (R)-5-((3-fluorophenyl) ethynyl)-N-(3-hydroxy-3-methylbutan-2-yl) picolinamide (VU0424465) was synthetized as detailed in the supplementary materials.

### Mutagenesis and Cell culture

All single point mutations of FLAG-SNAP-tagged mGlu_5_ receptor construct (Cisbio Bioassays) were generated using the Quick-change strategy (Agilent technologies) and verified by sequencing (Eurofins Genomics). HEK293 cells were cultured in Dulbecco’s modified Eagle’s medium (DMEM) supplemented with 10% fetal bovine serum (FBS) at 37 °C in a humidified 5% CO_2_ incubator. HEK293 cells were then transfected using Lipofectamine 2000 in a 96-well plate pre-coated with poly-dL-ornithine according to the manufacturer’s protocol. The DNA mixture included 40 ng of mGlu_5_ mutants, 50 ng of the glutamate transporter EAAC1 cDNA to avoid any influence of glutamate in the assay medium released by the cells and 60 ng of pRK6 for adjusting the final DNA concentration to 150 ng.

### Measuring cellular effects of mGlu_5_ G protein activation

Measurements of inositol phosphate (IP) accumulation and cyclic AMP in transfected cells were carried out in 96-well microplates using the IP-One and cAMP HTRF kits, respectively and according to the manufacturer’s recommendations (Cisbio Bioassays). After 48 h of transfection, HEK293 cells were incubated for two hours in DMEM Glutamax medium (Life Technologies) before measurements in order to reduce extracellular concentration of glutamate. After removing the medium, SNAP-mGlu_5_-∆856 agonist stimulation was performed by adding the ligand diluted to the desired concentration in the provided StimB buffer for the IP one kit or in DMEM medium complemented with cyclic nucleotide phosphodiesterase inhibitor Ro-20–1724 (50 μM) for the cAMP kit. The cells were then incubated for 30 min at 37 °C in a humidified 5% CO_2_ incubator. Cells were subsequently lysed by adding either IP1-d2 or cAMP-d2 conjugate followed by the terbium cryptate labelled anti-IP1 or anti-cAMP antibodies, respectively, both diluted in the provided lysis buffer. After one hour incubation at room temperature, the HTRF measurement was performed after excitation at 337 nm with fifty microseconds delay, terbium cryptate fluorescence and tr-FRET signals were measured at 620 nm and 665 nm, respectively, using a PHERAstar FS (BMG Labtech). Graphs were analysed using GraphPad Prism version 7.0 (GraphPad software, San Diego, CA). Functional concentration response data were fitted to a four parameters equation. The pEC_50_ values are expressed as the mean ± SEM.

### Detergent-solubilized receptor binding and thermostability measurements

After 48 h of transfection, HEK293 cells were incubated one hour at room temperature with the NAM [^3^H] MPEP at 80 nM (≈five times the K_D_) in a buffer containing 25 mM HEPES pH 7.4 and 400 mM NaCl. Membranes were then solubilized with 0.83% (w/v) MNG3, 0.042% (w/v) CHS for 1 h at 4 °C. Solubilized receptors (SNAP-mGlu_5_-∆856) were then incubated for 30 minutes at various temperatures ranging from 4 to 40 °C. The samples were then chilled at 4 °C for 5 min and the detergent solubilized ligand binding experiment performed as previously reported^22^. After mixing with liquid scintillation solution, bound radioligands were quantified using a MicroBeta liquid scintillation counter (Perkin Elmer). For insect cell expression, pellets from 1 mL Sf9 cells culture expressing mGlu_5_-∆856 were incubated for 1 hour at room temperature with 100 nM (0.5 µCi) of radiolgand [^3^H]- MPEP (ARC, inc) in binding buffer (25 mM HEPES (pH 7.4), 400 mM NaCl). Non-specific binding was determined by competition binding in the presence of 200 nM MPEP (Abcam). Solubilisation of the receptor was done by adding 0.83% (w/v) MNG3/0.083% (w/v) CHS and incubation for additional 1 h at 4 °C. Free and bound radioligand molecules were separated by a rapid filtration through gel filtration columns in batch mode as previously described^22^. Solubilized receptors were then incubated for 30 minutes at various temperatures ranging from 4 to 31 °C. 4 mL of scintillate solution was then added to the eluted solution and bound radioactivity was measured using liquid scintillation counter (Tri-Carb 2100TR, Packard). The counted radioactivity values were retrieved in disintegrations per minute (dpm). Specific binding was calculated by the difference between total binding and non-specific binding.

### Monitoring mGlu_5_ cell surface expression

Quantification of SNAP-tagged protein expression at the cell surface was monitored using SNAP-tag labelled with Lumi4-Tb. Briefly, cells were washed once with PBS and incubated 1 h at 37 °C with 100 nM of SNAP-Lumi4-Tb in Tag lite buffer (Cisbio Bioassays). Cells were then washed three times in PBS to eliminate unbound free dyes before fluorescence measurements. Lumi4-Tb fluorescence was measured, after excitation at 337 nm for 45 μs and emission at 620 nm, with a 50 μs applied delay on a PHERAstar FS (BMG Labtech).

### N-linked glycosylation site mutagenesis

N-linked glycosylation sites (NX (S/T)) were first predicted (Protter software) then mutated by a single substitution of the N residues to D making six distinct constructs: N88D (N1), N210D (N2), N378D (N3), N382D (N4), N445D (N5), and N734D (N6). These mutations were furthermore made in combination to generate the N89D/N210D/N445D (N1N2N5) and N89D/N210D/N378D/N382D/N445D (N1-5) constructs. All constructs were confirmed by sequencing (Eurofins Genomics). SNAP-mGlu_5_-∆856 mutants were expressed in the HEK293 cell line and labelled using SNAP-Red as described within previous section. Proteins were then extracted from membranes using 0.83% (w/v) MNG3/ 0.083% (w/v) CHS with mild shaking for 45 min at 4 °C. The solution was then centrifuged at 12,000 g for 30 min at 4 °C to eliminate the cells debris. 20 µL of supernatant supplemented with 10 mM dithiothreitol (DDT) reducing reagent were loaded after 5 min heating at 37 °C onto 4–20% Tris Glycine gels. Gels were scanned and fluorescently labelled SNAP-mGlu_5_-∆856 visualized after excitation in the far red (Odyssey imaging system).

### Generation of an mGlu_5_ construct optimized for Sf9 cells expression

The construct dedicated for purification optimisation in Sf9 cells was designed using overlapping PCR to have eGFP fusion protein at the C-terminus of the truncated human mGlu_5_ receptor after residue A856. The receptor sequence was subcloned into a modified pFastBac1 vector (Invitrogen), designated as pFastBac1, which contained an expression cassette with a GP64 peptide signal sequence from envelope surface glycoprotein of the *Autographa californica* nuclear polyhedrosis virus (AcNPV baculovirus) at the N-terminus and a TEV protease recognition site followed by the eGFP sequence and a 10× His tag, at the C-terminus of the receptor. The same construct was used to generate the mutated N-linked glycosylation sites (NX(S/T)) to Q (N89Q/N210Q/N378Q/N382Q/N445Q) by fragment gene synthesis (Eurofins Genomics). The construct designed for large-scale purification contained the GP64 peptide signal sequence, the FLAG (DYKDDDDK) and 10 × His tags, the precision protease recognition site (LEVLFQGP) at the N-terminus, respectively, followed by the human mGlu_5_ gene truncated at A856. All the constructs were subcloned into the pFastBac1 using PCR with primer pairs encoding restriction sites BamH1 at the 5′ and EcoR1 at the 3′ termini with subsequent ligation into the corresponding restriction sites found in the vector.

### Purification of mGlu_5_ produced in Sf9 cells

All the designed constructs were expressed in Sf9 cells grown in EX-CELL 420 medium (Sigma Aldrich) using the Bac-to-Bac Baculovirus expression system (Invitrogen). Cells were infected at a density of 3–4 × 10^6^ cells per mL with P2 baculovirus. Cultures were grown at 27 °C and collected 48 h post-infection then stored at −80 °C until use. Insect cell membranes from 4 L cultures were disrupted by thawing frozen cell pellets in a lysis buffer containing 25 mM HEPES (pH 7.4), 10 mM MgCl_2_, 20 mM KCl and complete protease inhibitor cocktail tablet (Roche). The supernatant was eliminated after two round of washing-centrifugation at 45,000 rpm with the same lysis buffer and an additional washing with a high salt buffer containing 25 mM HEPES (pH 7.4), 10 mM MgCl_2_, 20 mM KCl and 1 M NaCl. Membranes were resuspended with the lysis buffer supplemented with 40% glycerol then stored at −80 °C. Washed membranes were resuspended into a buffer containing 10 μM 2-methyl-6-(phenylethynyl)pyridine (MPEP, Abcam), 10 mM iodoacetamide (Sigma), complete protease inhibitor cocktail tablets and the additional protease inhibitors; bestatin, leupeptin, pefabloc and pepstatin (Roche). The mixture was then incubated at room temperature for 1 hour prior to solubilisation. The membranes were then solubilised in a buffer containing 25 mM HEPES buffer (pH 7.4), 0.4 M NaCl, 10% Glycerol, 0.5% (w/v) lauryl maltose neopentyl glycol (MNG3, Anatrace) and 0.05% (w/v) cholesteryl hemisuccinate (CHS, Sigma) for 1.5 hours at 4 °C, with shaking. The supernatant was then isolated by centrifugation at 45,000 rpm for 1 hour and supplemented with 10 mM imidazole (Sigma). For the mGlu_5_-∆856-eGFP construct, the supernatant was loaded onto a Ni^2+^ column (HisTrap HP, GE Healthcare) at 0.3 ml/min, overnight at 4 °C. After binding, the resin was washed with 20–30 column volumes of wash buffer (25 mM HEPES (pH 7.4), 400 mM NaCl, 10 μM MPEP, 10% (v/v) glycerol, 0.05 (w/v) MNG3, 0.005% (w/v) CHS, and 60 mM imidazole). However, for the mGlu_5_-∆856, supernatant was loaded onto Co^2+^ resin (Talon superflow, GE Healthcare) at 0.3 ml/min, overnight at 4 °C. The protein was then eluted with 25 mM HEPES (pH 7.4), 400 mM NaCl, 10 μM MPEP, 10% (v/v) glycerol, 0.05% (w/v) MNG3, 0.005% (w/v) CHS, and 250 mM imidazole. The eluted mGlu_5_-∆856 protein was incubated with an anti-FLAG affinity resin for 1 hour at 4 °C then washed with 10 column volumes of wash buffer (25 mM HEPES (pH 7.4), 150 mM NaCl, 10 μM MPEP, 0.01 (w/v) MNG3, 0.001% (w/v) CHS, and 2 mM calcium). The protein was eluted with a buffer containing 0.01% MNG3, 0.001% CHS, 25 mM HEPES pH 7.4, 0.15 M NaCl, 10 μM MPEP, 0.2 mg/ml FLAG peptide and 2 mM EDTA. The eluted fraction for both constructs were then concentrated using a Vivaspin 20 centrifugal concentrator 100 kDa cut-off (Sartorius), centrifuged for 10 min at 80,000 rpm to eliminate aggregates then loaded on to a size exclusion chromatography column (Superose 6 increase, GE Healthcare). The eluted fraction was further concentrated using a Vivaspin 6 centrifugal concentrator 100 kDa cut-off (Sartorius) and the protein concentration determined using the bicinchoninic acid (BCA) assay.

### Mass Spectroscopy

Protein purity was evaluated by Commassie blue-stained SDS-PAGE gels and the identity of bands revealed by mass spectroscopy. The SDS-PAGE gel showing two bands corresponding to the SEC peak were excised and destained. The protein was reduced with 10 mM TCEP in 25 mM NH_4_HCO_3_ solution at 56 °C for 30 min followed by 45 min incubation in 20 mM iodoacetamide for 45 min at room temperature. Gel pieces were then incubated with 20 µl trypsin (Promega) solution (19 ng/ul) to be digested overnight at 37 °C. The resulting fragments were then analysed by a MALDI-TOF TOF mass spectrometer.

### GTPγS binding

GTPγS binding experiments were carried out using the fluorescent Bodipy-FL GTPγS analog^26^. N-terminally His-Tagged Gα_s_ was expressed in *E. coli* and purified using nickel-nitrilotriacetic acid affinity (Ni-NTA) chromatography^36^. Gα_i2_ with an internal His-tag was expressed in *E. coli* and purified as described previously^37^. Soluble His_6_-tagged Gα_q_ was expressed in *Sf9* cells and purified using Ni-NTA affinity chromatography^38^. Finally, Gβ_1_ was expressed with the His_6_-Tagged Gγ_2_ in *Sf9* cells and the Gβγ dimer purified from membrane fractions using Ni-NTA chromatography combined to ion exchange chromatography^38^. Association of Bodipy-FL GTPγS to the G protein was monitored using a fluorescence spectrophotometer (Cary Eclipse, Varian) equipped with a Peltier-controlled temperature device, with the excitation wavelength set at 500 nm and the emission wavelength at 511 nm. Reaction conditions were 100 nM Gα_s_β_1_γ_2_, Gα_q_β_1_γ_2_ or Gα_i1_β_1_γ_2_, 100 nM Bodipy-FL GTPγS, and 20 nM mGlu_5_ receptor in detergent or reconstituted into 12% of DMPC: CHAPSO (bicelles) mixture at a 2.8:1. Fluorescence was monitored for 10 minutes at 15 °C after addition of the ligands (10 µM final concentration).

### Statistical analysis

Dunnett’s test as part of one-way ANOVA was used for multiple comparisons with a defined reference level. All graphs were analysed using GraphPad Prism version 7.0 (GraphPad software, San Diego, CA). Statistical significance was set as one, two, three and four stars to indicate P < 0.05, P < 0.01, P < 0.001 and P < 0.0001, respectively.

## Electronic supplementary material

Dataset 1
