# Supplementary material for: Direct coupling of detergent purified human mGlu5 receptor to the heterotrimeric G proteins Gq and Gs
Source: Sci Rep. 2018 Mar 13;8:4407. doi: 10.1038/s41598-018-22729-4 (PMC5849714; doi:10.1038/s41598-018-22729-4)
Supplement: Supplementary file 1 — Dataset 1 [file 41598_2018_22729_MOESM1_ESM.docx]

**Supplementary Information for**

**Direct coupling of detergent purified human mGlu_5_ receptor to the heterotrimeric G proteins Gq and Gs**

**Chady Nasrallah^1^, Karine Rottier^1^, Romain Marcellin^1^, Vincent Compan^1^, Joan Font^2^, Amadeu Llebaria^2^, Jean-Philippe Pin^1^, Jean-Louis Banères^3^, and Guillaume Lebon^1*^**

^1^ Institut de Génomique Fonctionnelle, Centre National de la Recherche Scientifique (CNRS), Institut National de la Santé et de la Recherche Médicale (INSERM), Université de Montpellier, F-34000 Montpellier (France).

^2^ MCS, Laboratory of Medicinal Chemistry, Institute for Advanced Chemistry of Catalonia (IQAC-CSIC), Barcelona, Spain.

^3^ Faculté de Pharmacie, Institut des Biomolécules Max Mousseron (IBMM), UMR 5247 CNRS-Université Montpellier-ENSCM, 15 Avenue C. Flahault, F-34093 Montpellier, France.

* Corresponding author: guillaume.lebon@igf.cnrs.fr

Supplementary Information include:

Supplementary figure 1 – Supplementary figure 5

Synthesis procedure for compound VU0424465

**SUPPLEMENTARY MATERIAL**

**
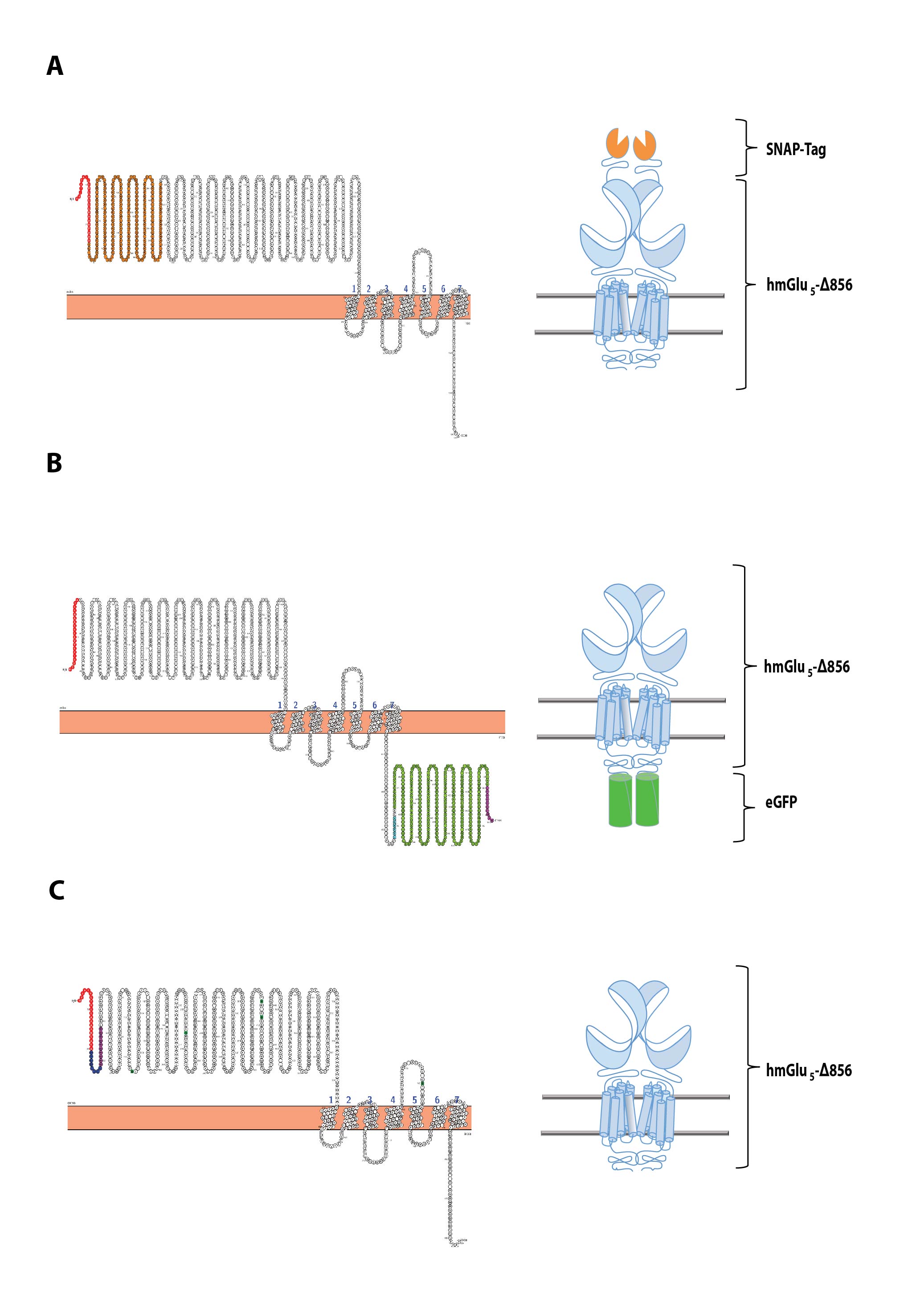
**

**Supplementary Fig. 1. Human mGlu_5_ constructs designed for expression in HEK293 and Sf9 cells.** (**A**-**C**) Starting from the N-terminus domain, (**A**) hmGlu_5_ construct (SNAP-hmGlu_5_-∆856) designed for HEK293 expression harbours, the wild type mGlu_5_ signal peptide (red) followed by an extracellular exposed SNAP-Tag fusion protein (orange) and a truncated C-terminus at A856 where a stop codon is inserted; (**B**) The Sf9 hmGlu_5_ designed construct for purification optimization (hmGlu_5_-∆856-eGFP) harbours, the GP64 peptide signal sequence (red), the A856 truncated version of the hmGlu_5_ gene followed by the intracellular eGFP fusion protein (green) and a 10× His tag (purple), at the C-terminus of the receptor; (**C**) The Sf9 hmGlu_5_ designed construct for large scale purification (hmGlu_5_-∆856) harbours, the GP64 peptide signal sequence (red), followed by the Flag (blue) and the 10×His (purple) tags and a truncated version of the hmGlu_5_ gene where a stop codon was inserted at position 856. All the plots were generated using Protter^1^. A schematic representation of the three constructs is shown at the right of each snake plot.

**
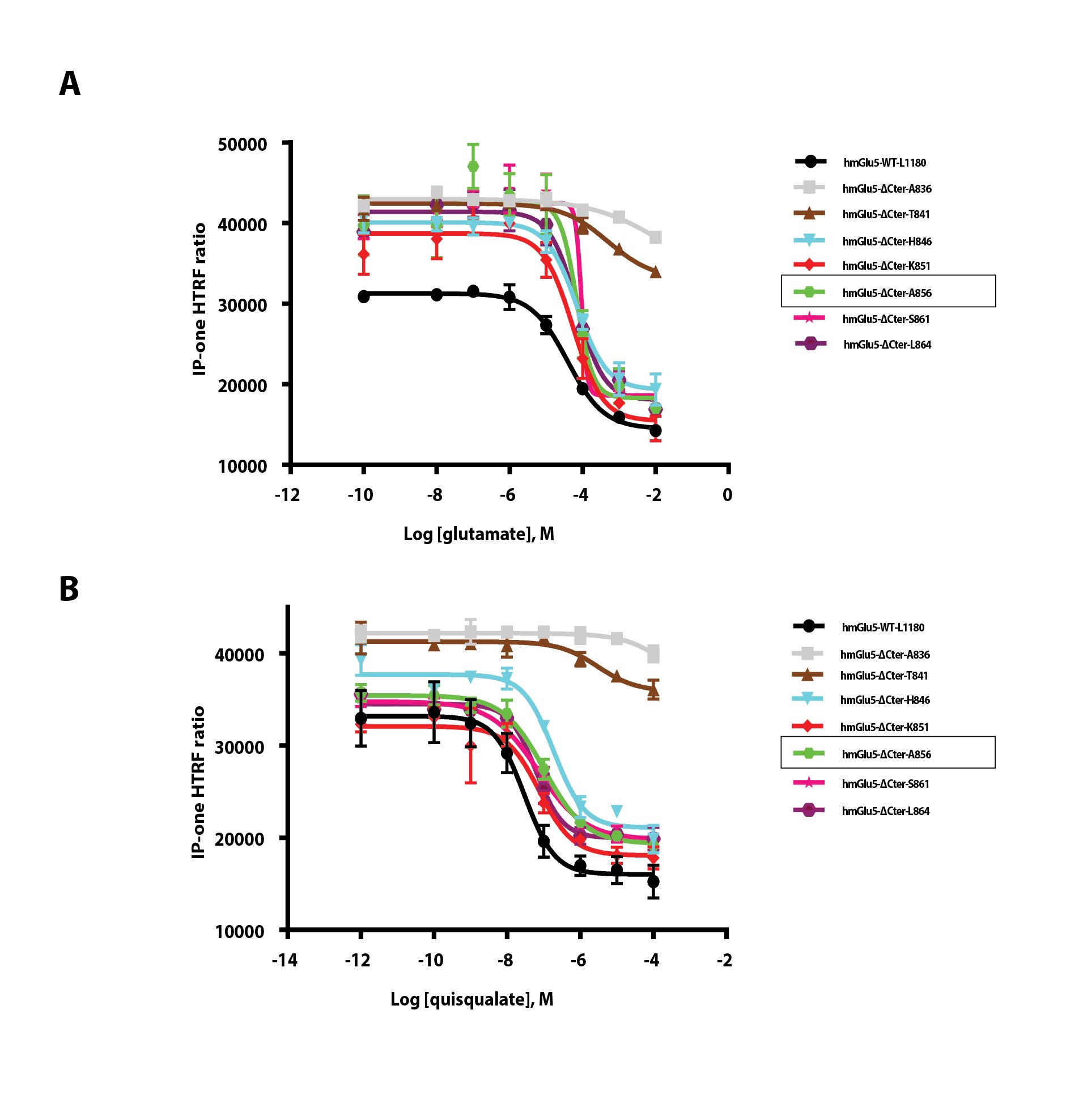
**

**Supplementary Fig. 2. Monitoring IP-one release in HEK293 cells for C-terminus truncated hmGlu_5_ using IP-One assay. (A, B)** IP-one release is measured for seven truncated constructs at the C-terminus of hmGlu_5_ following an increased concentration of agonists, glutamate (**A**) and quisqualate (**B**). Data points represent the mean ± S.E.M. of at least two independent experiments.

**
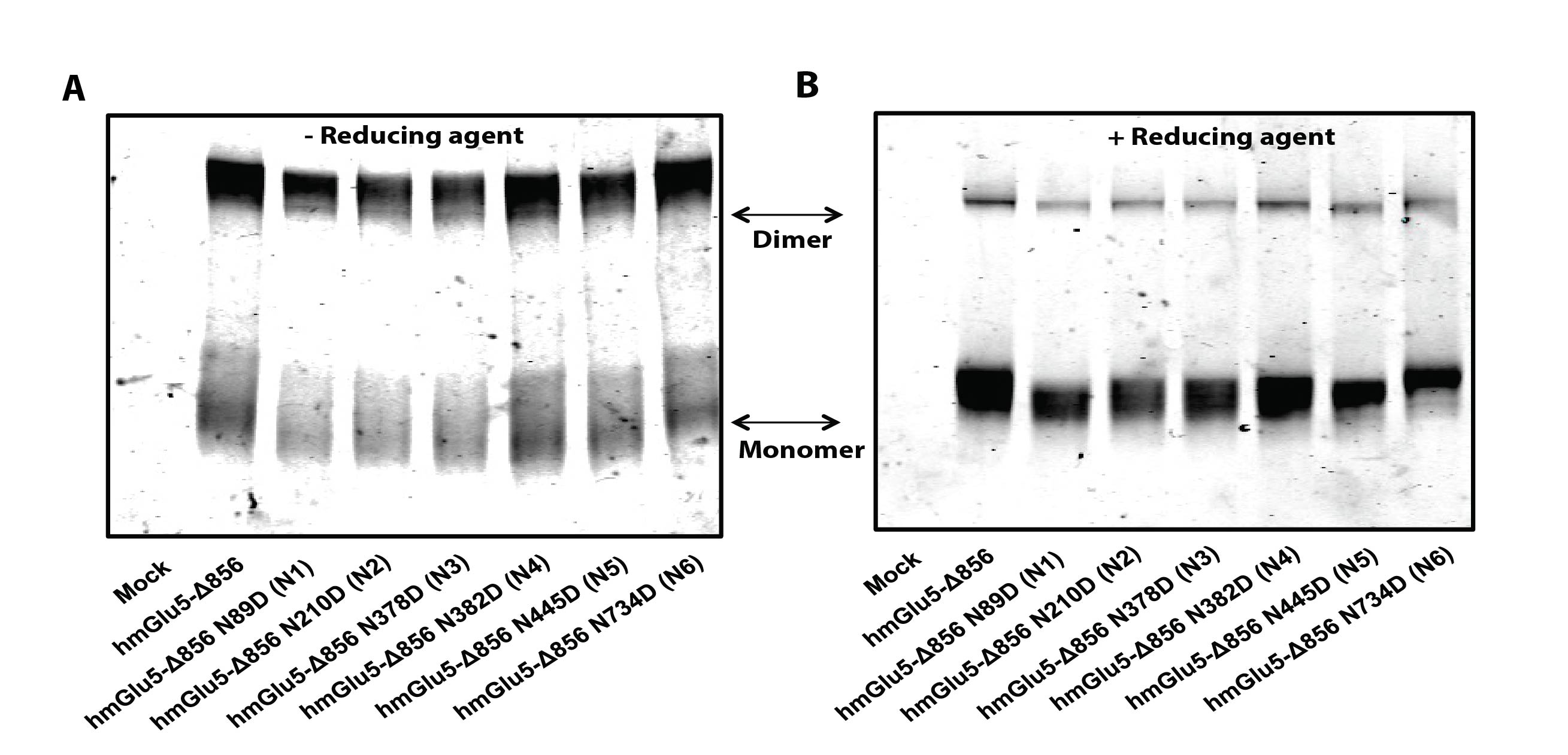
**

**Supplementary Fig. 3. SDS PAGE analysis of the HEK293 expressed SNAP-hmGlu_5_-∆856 that carries the single N-linked mutations.** (**A, B**) The SDS PAGE gel (**A**) reveals migration of intense fractions of dimers and light fraction of monomers in denaturating conditions and in the absence of DTT for both, the wild-type hmGlu_5_-∆856 and the engineered hmGlu_5_-∆856 N/D mutants. The SDS PAGE gel (**B**) reveals migration in denaturating conditions of the same samples in A, but in the presence of 10 mM DTT. To note the same gel (right) was used to illustrate Fig. 2, B.


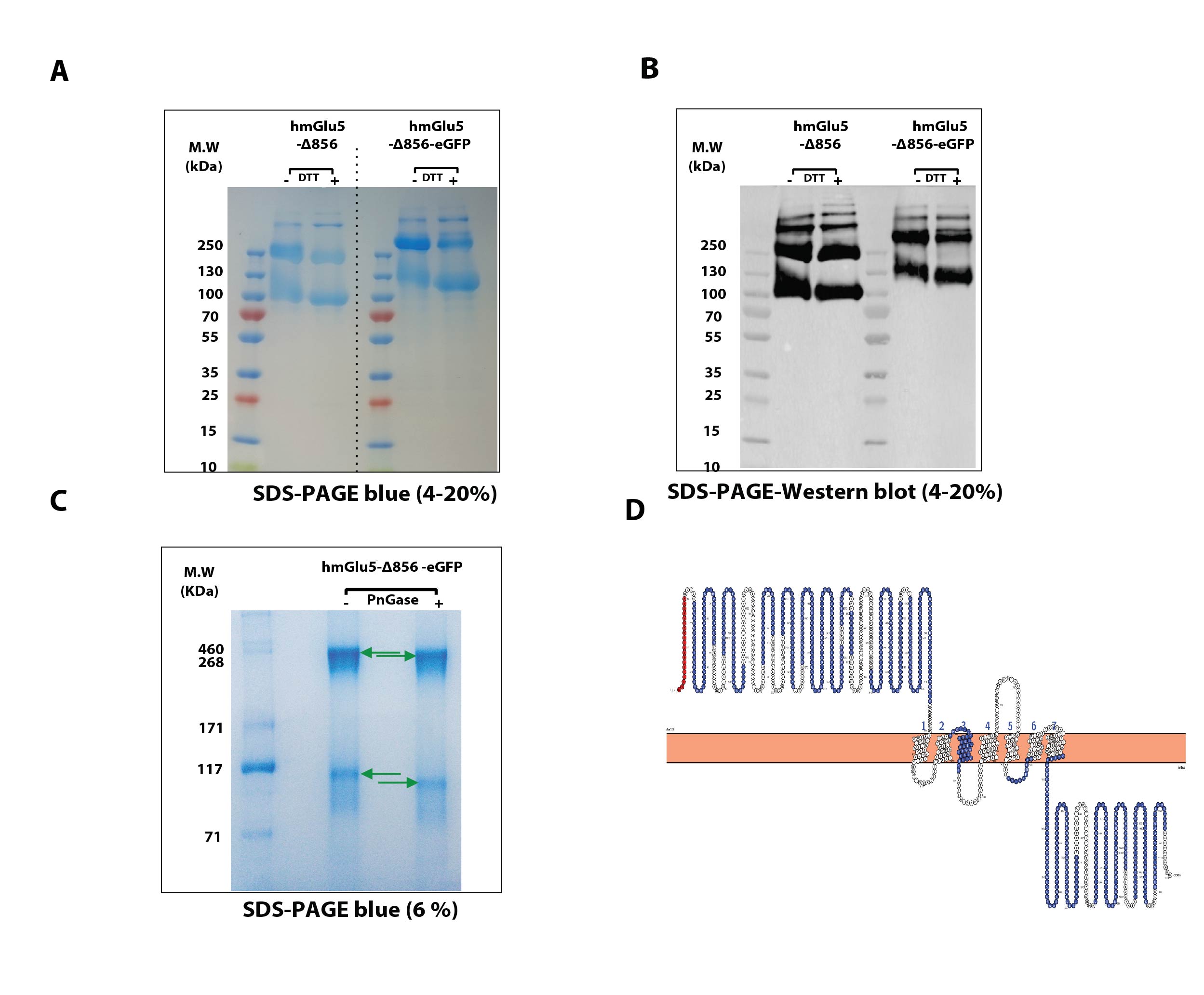


**Supplementary Fig. 4. Western blot and mass spectroscopy confirm hmGlu_5_ identity produced in Sf9 cells.** (**A**) SDS PAGE (4-20%) of the purified hmGlu_5_ with and without eGFP fusion protein from Sf9 cells, in the presence (+) or in the absence (-) of DTT in the sample. Two bands are present and correspond to the receptor dimeric (upper) and monomeric (lower) states. (B) Bands identity was furthermore confirmed by western blot using monoclonal anti-Flag antibody for mGlu_5_-∆856 and anti-His antibody for hmGlu_5_-∆856-eGFP constructs. (**C**) SDS PAGE (6%) reveal migration of hmGlu_5_-eGFP to a lower molecular weight (green arrows) for both forms, upper and lower bands, after PNGase enzyme treatment. (**D**) Both mGlu_5_-∆856-eGFP bands, revealed on SDS PAGE gel, were analysed by mass spectroscopy and identified as mGlu_5_-∆856-eGFP with almost 60% coverage. Trypsin digested segments are highlighted in blue. The plot was generated using Protter^1^.

**
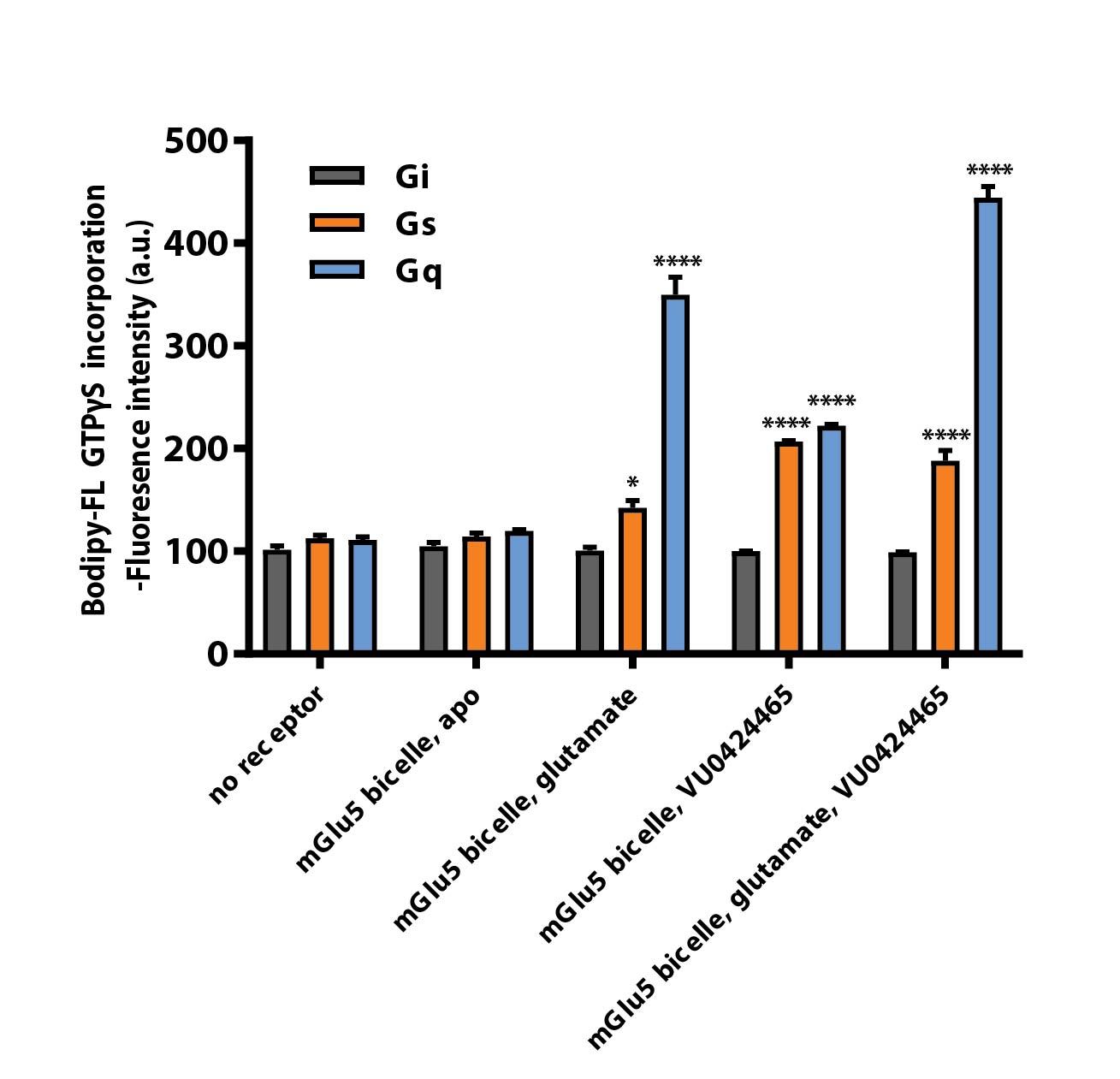
**

**Supplementary Fig. 5. Reconstitution of mGlu_5_-Δ856 in bicelles increased mGlu_5_-dependent Gα_q_ and Gα_s_ activation level.** The bodipyl FL GTP**γ**S fluorescence was monitored for purified mGlu_5_-Δ856 reconstituted in bicelles after binding of both glutamate and/or VU0424465. Gα_i_ was measured as internal control and shows no response for glutamate alone or for both glutamate and VU0424465. The fluorescence baseline was estimated based on the receptor alone without any ligands (Apo). Data points represent average of three independent measurements with SEM. Dunnett’s test as part of one-way ANOVA was used for comparison with the apo condition set as reference level.

**Synthesis and chemical characterization of positive allosteric modulator compound VU0424465.** Synthesis of compound **7** was prepared as shown in *scheme S6,* all compounds were described previously by Conn and collaborators^2^. Starting from commercial (*R*)-Methyl 3-((tert-butoxycarbonyl)amino)propanoate (**1**) it was reacted with MeMgBr (5.0 equiv) yielding tertiary alcohol **2** in 80% yield, which was deprotected in the presence of TFA/DCM (1:2) to give the aminoalcohol **3** as its TFA salt, in 97% yield. On the other hand, Sonogashira coupling between bromopicolinic acid (**4**) and 1-ethynyl-3-fluorobenzene (**5**) to yield the carboxylic acid **6** in 58% of yield. Finally, compound **7** was afforded by acylation between the acid chloride of **6** and the removing of the TFA salt with triethylamine generating both of them *in-situ* in 99% yield.

Scheme 6. Synthesis of compound **7**. Reagents and conditions: a) MeMgBr (5.0 equiv), THF, 0 ºC to rt, overnight, 80%; b) TFA/ CH_2_Cl_2_ (1:2), 0 ºC, 1h, 97%; c) CuI, PdCl_2_(PPh_3_)_2_, Et_3_N, DMF, 50 ºC, Overnight, 58%; d) 1. SOCl_2_, CH_2_Cl_2_; 2. Et_3_N, DCM, reflux, 99%.

**(*R*)-*tert*-Butyl (3-Hydroxy-3-methylbutan-2-yl)-carbamate** (**2**): (*R*)-Methyl 3-((tert-butoxycarbonyl)amino)propanoate **1** (2 g, 9.8 mmol, 1 equiv) was dissolved in THF (50 mL, 0.2 M) and cooled to 0 ºC. MeMgBr (16.4 mL, 49.2 mmol, 5 equiv, 3.0 M sol in Et_2_O) was added slowly, and the reaction was allowed to warm to room temperature and stirred overnight. The reaction was then quenched carefully with NH_4_Cl (aq sat) and extracted with EtOAc (3 x 75 mL). The organic layer was dried with Na_2_SO_4_ and concentrated. The crude oil was purified by silica gel chromatography eluting with Hexane/EtOAc 4:1. (R)-*tert*-Butyl (3-Hydroxy-3-methylbutan-2-yl)-carbamate was isolated as a clear oil (1.6 g, 80%)^2^. ^1^H NMR (400 MHz, Chloroform-*d*) δ 4.72 (s, 1H), 3.58 (s, 1H), 2.14 (s, 1H), 1.43 (s, 9H), 1.21 (s, 3H), 1.16 (s, 3H), 1.12 (d, *J* = 6.8 Hz, 3H).

**(*R*)-3-Amino-2-methylbutan-2-ol** (**3**): (*R*)-*tert*-Butyl (3-Hydroxy-3-methylbutan-2-yl)-carbamate (**2**) (1 equiv) was dissolved in CH_2_Cl_2_ (0.1 M) and cooled to 0 ºC. Trifuoroacetic acid (0.2 M) was added, and the reaction mixture was stirred for 1 hour. The starting material was determined to be consumed by TLC, and the reaction mixture was concentrated. The resulting TFA salt of (R)-3-Amino-2-methylbutan-2-ol (**3**) was afforded as red-brown oil and used in next step without further purification.

**5-((3-Fluorophenyl)ethynyl)picolinic Acid** (**6**): In a 100 mL round-bottom flask, 5-bromopicolinic acid (**4**) (1 g, 4.95 mmol, 1 equiv), PdCl_2_(PPh_3_)_2_ (104 mg, 0.149 mmol, 0.03 equiv), and CuI (28 mg, 0.149 mmol, 0.03 equiv) were combined under argon atmosphere and degassed by Ar/vacuum cycles. The solid mixture was disolved in DMF (6 mL, 0.8 M). 3-Fluorophenylacetylene (**5**) (687 μL, 5.94 mmol, 1.2 equiv) was added, followed by Et_3_N (4.14 mL, 29.7 mmol, 6 equiv). The reaction mixture was heated to 50 ºC overnight after which the reaction was determined to be complete by TLC. The crude reaction mixture was diluted with EtOAc (50 mL) and H_2_O (50 mL). After separating the organic layer, the aqueous layer was washed with EtOAc (2 x 25 mL). The aqueous layer was the acidified until pH = 2 with 2 M HCl and extracted with EtOAc (3 x 50 mL). The organic layer was dried with Na_2_SO_4_, concentrated, and used without further purification. The product was isolated as a white solid (700 mg, 58%)^2^. ^1^H NMR (400 MHz, Methanol-*d*_4_) δ 8.83 – 8.79 (m, 1H), 8.18 (dd, *J* = 8.1, 0.9 Hz, 1H), 8.12 (dd, *J* = 8.1, 2.0 Hz, 1H), 7.46 – 7.40 (m, 2H), 7.38 – 7.32 (m, 1H), 7.24 – 7.15 (m, 1H).

**(*R*)-5-((3-fluorophenyl)ethynyl)-*N*-(3-hydroxy-3-methylbutan-2-yl)picolinamide** (**7**): First, 5-((3-Fluorophenyl)ethynyl)picolinic Acid (**6**) (700 mg, 2.9 mmol, 1 equiv) was solved in SOCl_2_ (10.5 mL, 145 mmol, 50 equiv) and the reaction mixture was heated at 80 ºC during 2 hours. Afterwards, the reaction mixture was concentrated and the residue disolved in CH_2_Cl_2_ (10 mL, 0.3 M). A mixture of the TFA salt of (R)-3-Amino-2-methylbutan-2-ol (**3**) (630 mg, 2.90 mmol, 1 equiv) and triethylamine (8.1 mL, 58 mmol, 20 equiv) in CH_2_Cl_2_ (5 mL, 0.6 M) was added to the first solution carefully, and the reaction mixture was stirred at room temperature overnight. The reaction mixture was washed with 1 M HCl, followed by aq. Sat. NaHCO_3_. The organic layer was separated, dried over Na_2_SO_4_ and concentrated. The brown crude was purified by silica gel chromatography eluting with Hexane/EtOAc 1:4 isolating a white solid (937 mg, 99%). ^1^H NMR (400 MHz, Chloroform-*d*) δ 8.64 (dd, *J* = 2.1, 0.9 Hz, 1H), 8.17 (dt, *J* = 8.1, 1.9 Hz, 2H), 7.93 (dd, *J* = 8.1, 2.1 Hz, 1H), 7.36 – 7.30 (m, 2H), 7.27 – 7.21 (m, 1H), 7.13 – 7.04 (m, 1H), 4.12 (dd, *J* = 9.2, 6.9 Hz, 1H), 2.64 (s, 1H), 1.40 – 1.18 (m, 11H). HPLC-Ms. >93% purity. HRMS Calculated for C_19_H_20_N_2_O_2_F: 327.1509; Found : 327.1506.

**^1^H-NMR spectra of key compounds 2, 6, and 7**


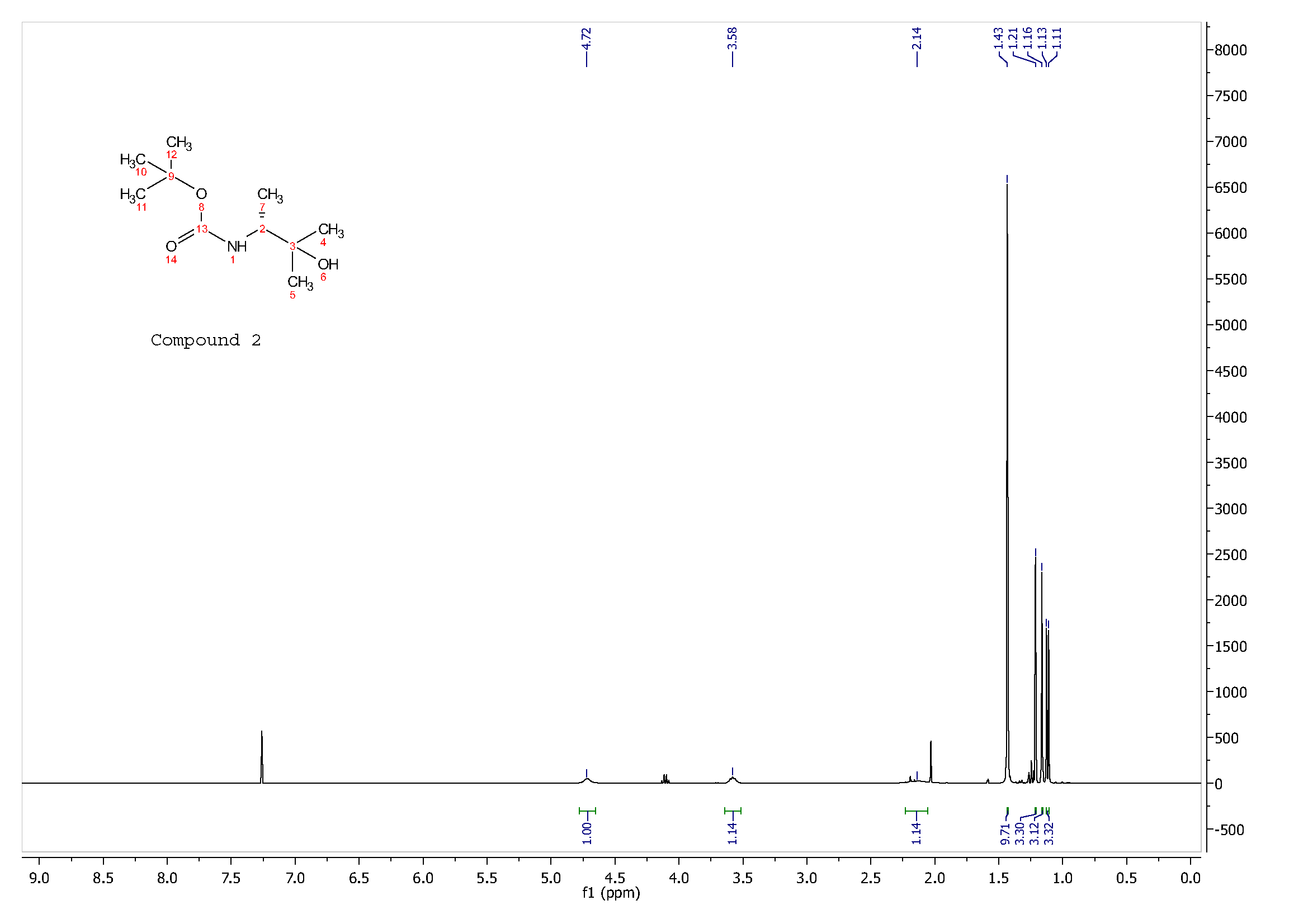

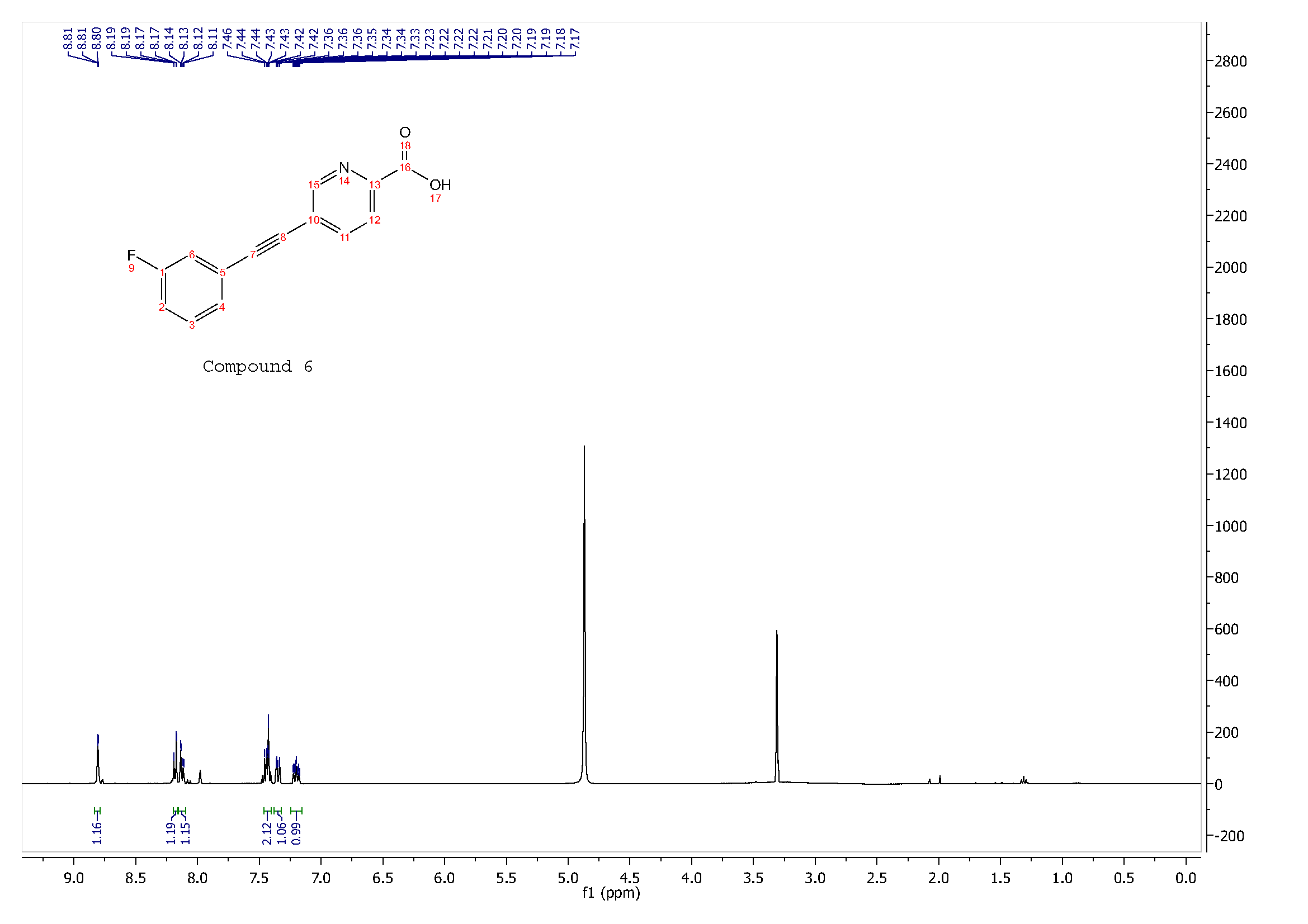


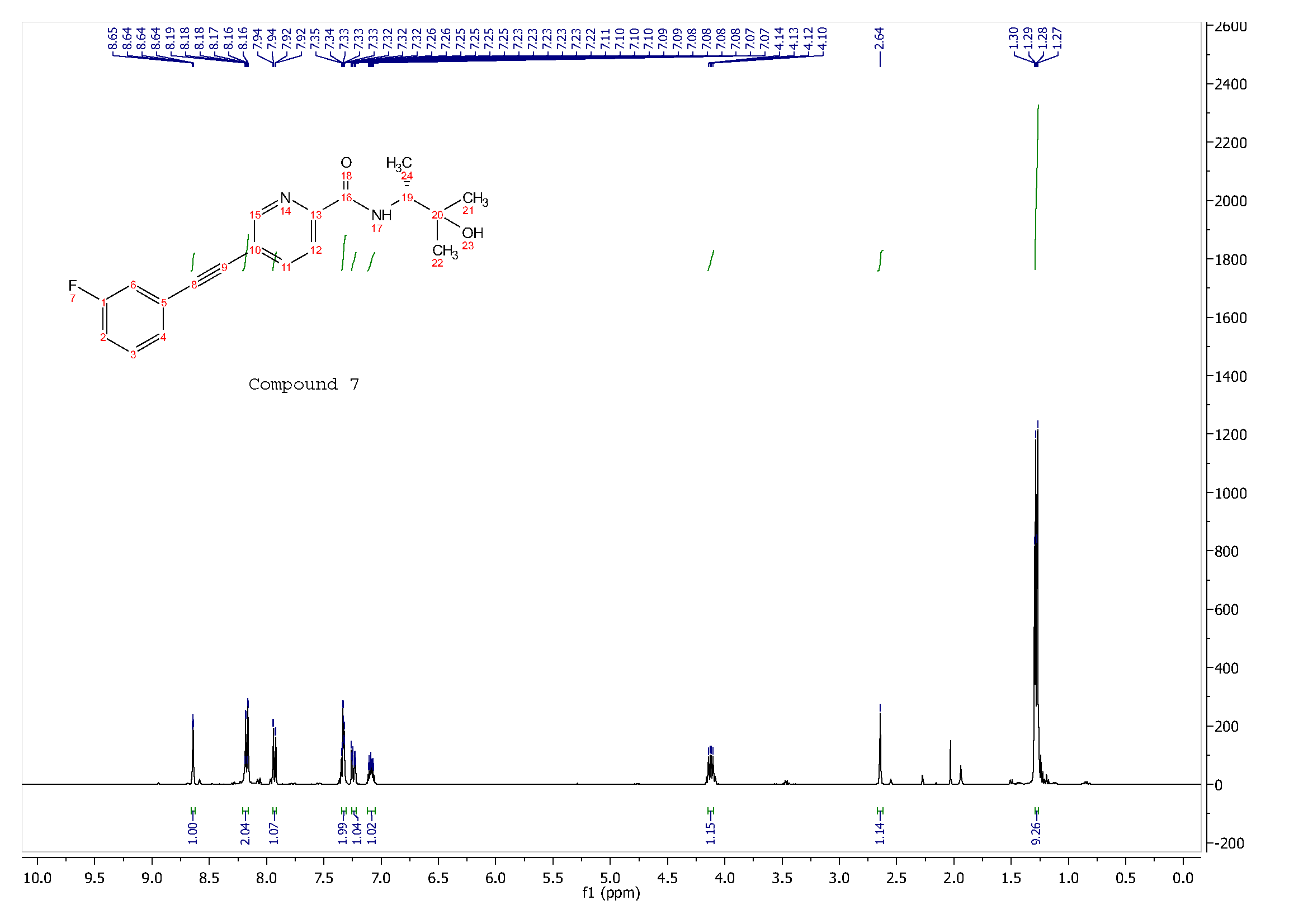


**Analytical data for compound 7**

HPLC/MS


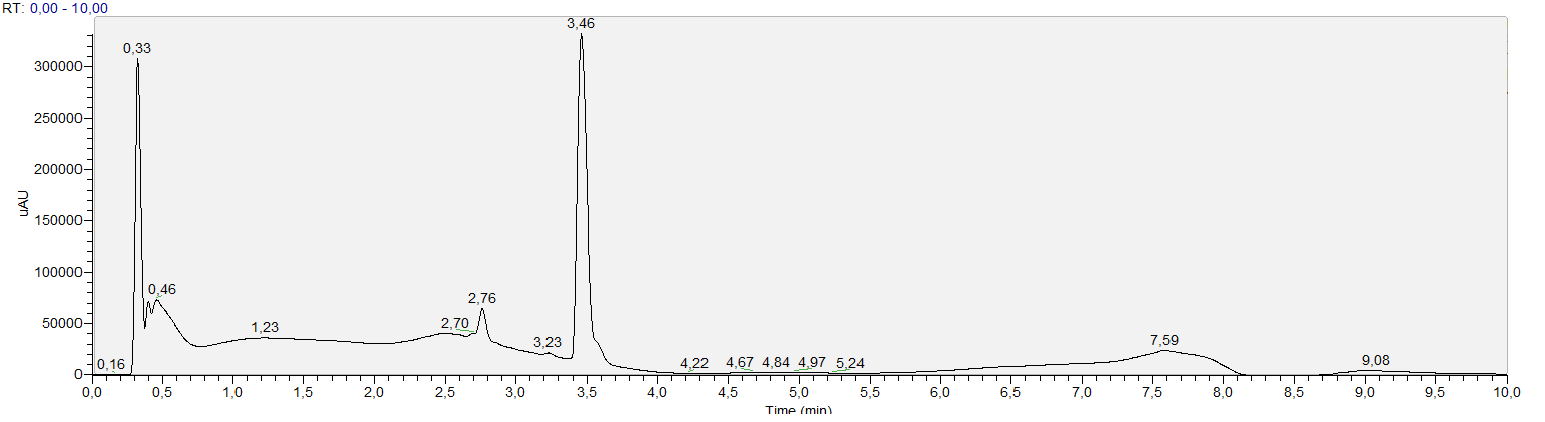


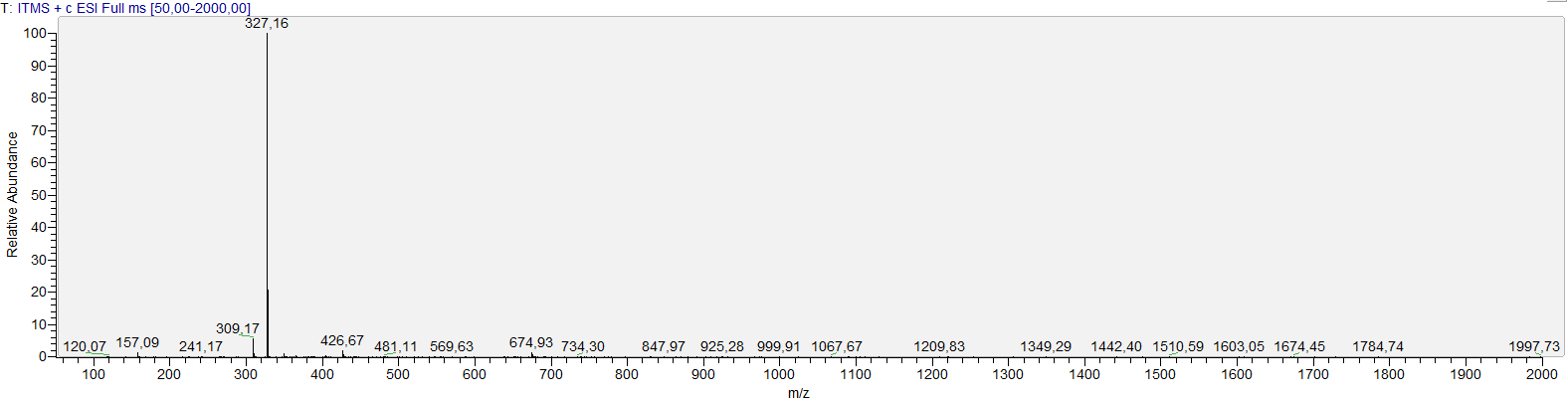


| Apex RT | Start RT | End RT | Area | %Area |
| --- | --- | --- | --- | --- |
| 2,76 | 2,67 | 2,84 | 116186,147 | 6,80 |
| 3,46 | 3,37 | 3,71 | 1592246,950 | 93,20 |

HRMS


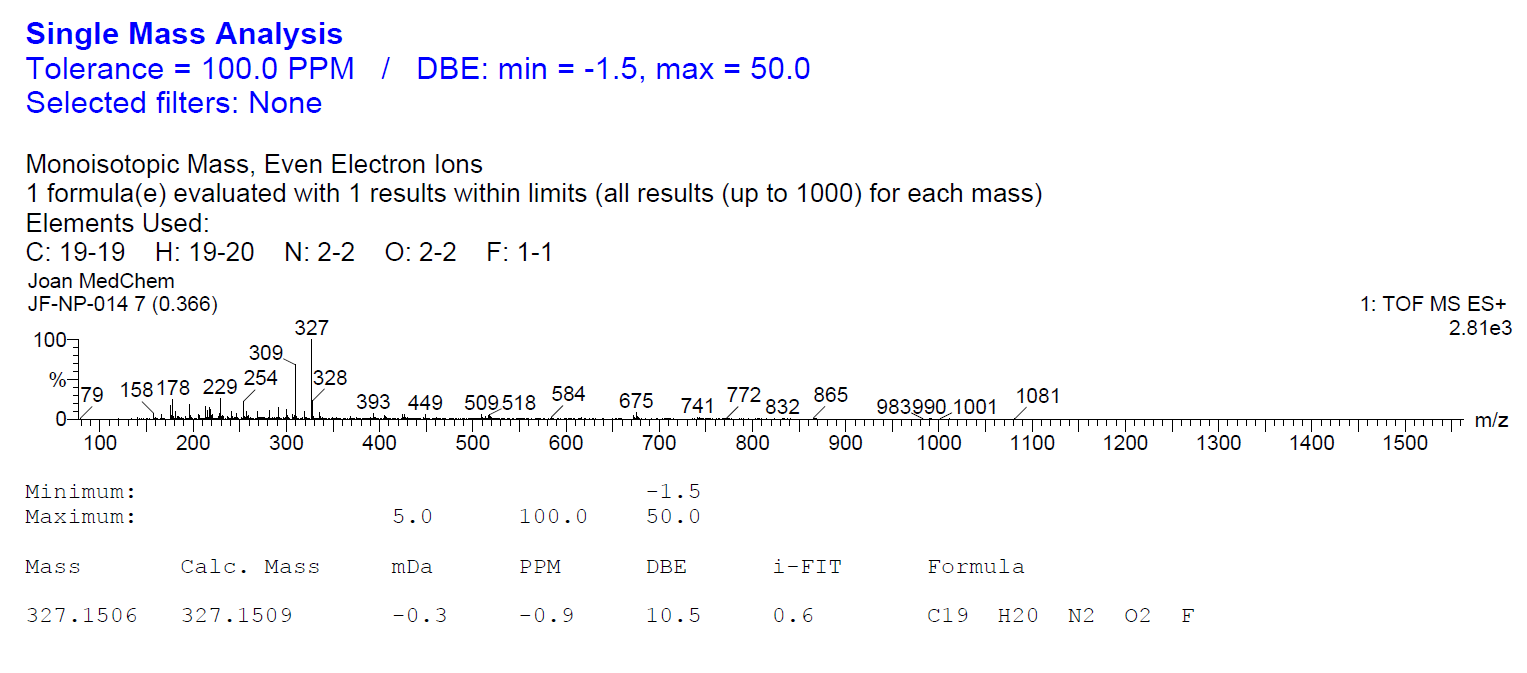


**Bibliography**

1. Omasits, U., Ahrens, C.H., Muller, S. & Wollscheid, B. Protter: interactive protein feature visualization and integration with experimental proteomic data. *Bioinformatics* **30**, 884-886 (2014).

2. Turlington, M. *et al.* Exploration of Allosteric Agonism Structure-Activity Relationships within an Acetylene Series of Metabotropic Glutamate Receptor 5 (mGlu(5)) Positive Allosteric Modulators (PAMs): Discovery of 5-((3-Fluorophenyl)ethynyl)-N-(3-methyloxetan-3-yl)picolinamide (ML254). *Journal of Medicinal Chemistry* **56**, 7976-7996 (2013).
